# Precursor Inhomogeneities Influence the Properties of Multimetal Oxides as Shown for LiNi_0.5_Mn_1.5_O_4_ Derived from Hydrothermally Synthesized Precursors

**DOI:** 10.1002/advs.202508174

**Published:** 2025-07-27

**Authors:** Simon Schauer, Bastian Beitzinger, Gregor Neusser, Christine Kranz, Mika Lindén

**Affiliations:** ^1^ Inorganic Chemistry II Ulm University Albert‐Einstein‐Allee 11 89081 Ulm Germany; ^2^ Institute of Analytical and Bioanalytical Chemistry Ulm University Albert‐Einstein‐Allee 11 89081 Ulm Germany

**Keywords:** correlative microscopy, hydrothermal synthesis, LMNO, mixed metal oxide, Raman imaging

## Abstract

Co‐precipitation is an attractive means for synthesizing mixed metal oxides, a material class with a wide range of application prospects. Often soluble metal salts are precipitated as metal hydroxides or ‐carbonates, which subsequently are converted to the corresponding oxide(s) upon thermal treatment. The thermal treatment step is used to induce compositional homogeneity on the atomic level. In order to shed some more light on this effect, detailed analyses of a mixed Li‐Ni‐Mn‐oxide with the target composition LiNi_0.5_Mn_1.5_O_4_ (LNMO), which is a promising cathode material for rechargeable Li‐ion batteries are performed. The first synthesis step is the precipitation of a Ni‐Mn‐carbonate, which is subsequently thermally lithiated and transformed into an oxide. A combination of X‐Ray diffraction (XRD), Raman microscopy, scanning electron microscopy coupled with energy dispersive X‐Ray spectroscopy (EDX) in combination with elemental analysis, as well as electrochemical characterization, revealed that chemical inhomogeneities present already in the precursor particles have a direct influence on the electrochemical performance of the final product. The broader implications of these results in relation to co‐precipitation‐based syntheses of functional materials are discussed, with special focus on the importance of the particle size distribution of the secondary particles.

## Graphical abstract

Correlative Raman and scanning electron microscopies are used for identifying compositional differences on the micrometer length scale in LiNi_0.5_Mn_1.5_O_4_ cathode materials, and it is shown that these compositional differences can be linked to the electrochemical signature of the material.

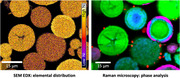

## 1 Introduction

Mixed metal oxides (MMOs), typically containing one or more transition metals, have a variety of applications due to their unique properties, such as high thermal stability, catalytic activity, electrical conductivity, and magnetic properties. Prominent examples include Cu‐Zn‐Al‐containing oxides used as catalysts in methanol synthesis,^[^ ^1^ ^]^ Li‐Ni‐Mn‐CoO_2_^[^ ^2^, ^3^, ^4^ ^]^ or LiNi_0.5_Mn_1.5_O_4_^[^ ^5^, ^6^ ^]^ (LNMO) used as cathode materials in rechargeable Li‐ion batteries, and Bi‐Ti‐oxides^[^ ^7^ ^]^ used in ferroelectric applications. A common method used for the synthesis of MMOs is co‐precipitation,^[^ ^8^, ^9^, ^10^ ^]^ where a stoichiometric mixture of the metal salts or ‐alkoxides are dissolved in a solvent, typically water, and where the metal ions are then precipitated out to form hydroxides^[^ ^11^ ^]^ or carbonates^[^ ^12^, ^13^ ^]^ through addition of a base or a carbonate source when the solubility product concentration, or if under kinetic control, the critical supersaturation concentration, of the system is reached.^[^ ^14^, ^15^, ^16^ ^]^ As the metals typically have differing solubility products, batch type co‐precipitation should inevitably lead to compositional gradients in the resulting precursor particles, and possibly even to compositional differences from particle to particle. The obtained precursors are then isolated through filtration, dried, and subsequently calcined at elevated temperatures. The high‐temperature treatment is important not only for converting the hydroxides or carbonates into the corresponding oxides, but also in many cases to ensure a homogeneous mixing of the metals through temperature‐induced metal ion mobility if the components are mutually miscible.

This redistribution of the metals during tempering is important, as in most cases the different metal hydroxides/carbonates have different values of their solubility products, which may lead to compositional inhomogeneities in the precipitate.^[^ ^14^ ^]^ Nanoscale inhomogeneities can have important influences on the performance of functional materials, as recently shown for Co_2_FeO_4_ spinel and their activity as catalysts for the oxygen evolution reaction.^[^ ^17^ ^]^ Upon co‐precipitation from a solution containing two precursors exhibiting different values of the solubility products, particles having a core rich in one component and a shell rich in the other could be expected to form. This was observed in a study by Liu et al.^[^ ^13^ ^]^ related to the synthesis of LiNi_0.5_Mn_1.5_O_4_, an important cathode material for Li‐batteries,^[^ ^18^, ^19^ ^]^ where the precipitated mixed metal carbonate precursor microparticles consisted of an Mn‐rich core and which had a rim of essentially pure NiCO_3_. In the study by Liu, upon thermal lithiation followed by calcination under air, phase‐pure LNMO was stated to form. This is of special interest, as the Ni from the rim area needs to be homogenously distributed throughout the whole particle to achieve the stoichiometric composition needed for phase‐pure LNMO spinel. The distribution of Ni is especially important for LNMO materials, as in fully stoichiometric LNMO, only Ni(II) is electrochemically active, leading to a single voltage plateau ≈4.7 V versus Li/Li^+^, while the Mn(IV) atoms are electrochemically inactive. If there are less than stoichiometric amounts of Ni(II) present in the spinel lattice, the loss of Ni(II) needs to be compensated via reduction of Mn(IV) to Mn(III), which leads to an additional voltage plateau ≈4.1 V versus Li/Li^+^, resulting from the Mn(III)/Mn(IV)‐transition.^[^ ^20^ ^]^ Therefore, this synthesis appears to be a promising model system for the study of the influence of precursor‐level compositional inhomogeneities on the properties of the final material. Furthermore, the fact that the LNMO synthesis in this case is carried out using carbonate precursors, makes it possible to apply Raman microscopy for the study of spatial compositional differences in the precursor, as discussed in more detail below.

Furthermore, there are two existing modifications of the LNMO spinel material: disordered LNMO (d‐LNMO) spinel crystallizes in a cubic face‐centered spinel lattice (*Fd3̄̄m*), while the ordered LNMO (o‐LNMO) spinel has a simple, primitive cubic spinel structure (*P4_3_32*). In the disordered structure, nickel and manganese atoms are randomly distributed on the octahedral 16c sites. Lithium atoms occupy tetragonal 8c sites, and oxygen atoms occupy the 32e positions. In contrast, in stoichiometric ordered LNMO spinel, nickel and manganese atoms occupy fixed positions. Manganese atoms exclusively occupy the 12c positions, while nickel atoms exclusively occupy the 4a position. Lithium atoms are located on 4c sites, and oxygen atoms are distributed on the 8c and 24e sites.^[^ ^21^ ^]^ While both modifications have a similar lattice structure and are hardly distinguishable via classic bulk characterization methods like PXRD, their Raman spectra are drastically different due to the higher degree of cation ordering. Furthermore, Raman microscopy can be used for evaluation of the stoichiometry of mixed Ni‐Mn‐carbonate precursors.^[^ ^22^, ^23^ ^]^

Thus, this synthesis can serve as a suitable model system for investigating the occurrence of compositional inhomogeneities on an atomic and microscopic scale in MMOs prepared by batch co‐precipitated precursors, and more specifically, on the influence of compositional homogeneities on the electrochemical performance of LNMO cathode materials. Thus, the precursors were tempered with typical thermal treatments used to achieve the d‐ and o‐LNMO modifications, respectively, to reveal differences in the metal distribution afterwards. In an additional step, the d‐LNMO modification was treated with the program for o‐LNMO to investigate the possibility of a purely thermal transformation of d‐LNMO into o‐LNMO. Subsequently, to allow for a structural and compositional characterization on the precursor and functional material level, a combination of scanning electron microscopy – energy dispersive X‐Ray spectroscopy (SEM‐EDX) and Raman spectroscopy/microscopy was applied using a correlative approach. This way, the results of all three measurements performed on exactly the same cross‐sectioned particles can be combined to reveal chemical, structural and compositional information, which in turn should enable us to explain the material's electrochemical performance. The electrochemical properties were evaluated by charge‐discharge measurements and derived d*Q*/d*U*‐plots in a half‐cell configuration against Li for the final LNMO materials.

## 2 Results

### 2.1 Synthesis of Mixed Carbonate Precursors

**Figure 1:**
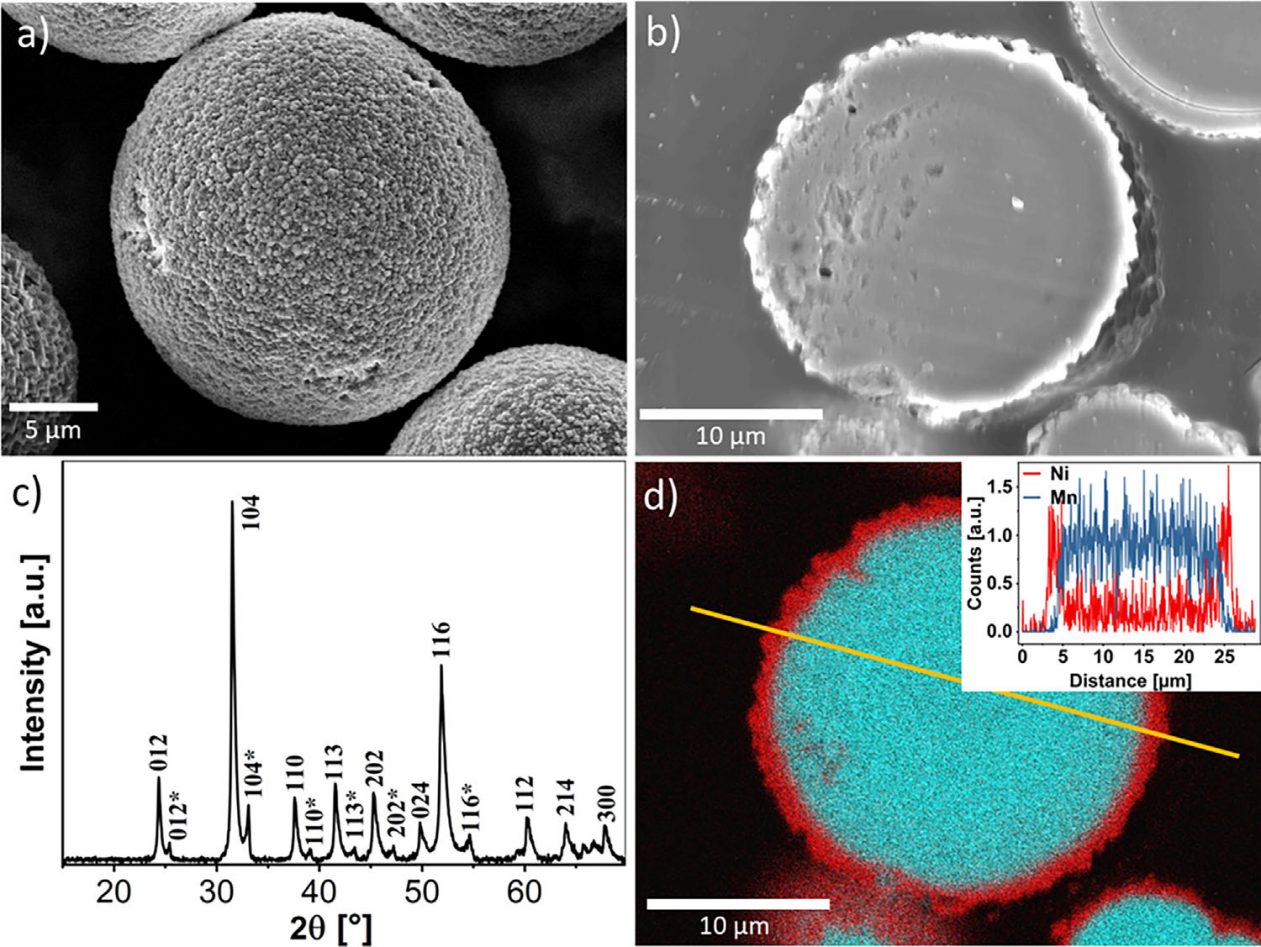
Exemplary SEM images of a) the mixed metal carbonate (Mn_0.75_Ni_0.25_) CO_3_ precursor secondary particles with image derived particle size distribution plot (insert) and b) cross‐section of a precursor particle embedded in EPON and cut by microtome. c) The PXRD diffractogram of the precursor exhibits the typical reflexes for mixed (Mn,Ni)CO_3_ and NiCO_3_ (the latter marked with asterisks). d) EDX image of the particle cross‐section shown in b) displaying the distribution of Ni (red) and Mn (cyan) within the particles together with an intensity line plot of the two elements across the sample (see insert in d)).

Mn/NiCO_3_ microparticles were hydrothermally synthesized according to the procedure published by Liu with slight modifications. Instead of using urea as the sole source for carbonate, a mixture of urea and dimethyl carbonate was used, as this, together with an addition of a small amount of the CTAB lead to a better dispersed product in our reactor setup. In agreement with the original synthesis, spherical, secondary particles with a mean particle diameter of ≈19 µm (±4 µm, n  =  398, particle sizes ranged from 10 to 30 µm) were obtained, as shown in **Figure** **1**a.

The 10–30 µm sized secondary particles, which will be referred to as “particles” in the following if not mentioned otherwise, consisted of agglomerated primary nanoparticles, as seen in Figure 1a. ICP‐MS revealed an overall composition of (Mn_0.75±0.01_Ni_0.25±0.01_CO₃), aligning closely with the desired Ni/Mn‐ratio of the prospective LNMO product. Cross‐sectional analysis of the particles revealed a Mn‐rich interior and an Ni‐rich rim (Figure 1b,d). XRD Rietveld analyses (R_wp_ = 7.963, R_p_ = 5.356, Goodness of fit = 3.482) confirmed that the particles consisted a mixture of Mn/NiCO_3_ (Figure 1c), and the primary phase had a hexagonal crystal system assignable to the space group *R3̄̄c* ^[^ ^24^ ^]^ (a = b = 4.7719 Å and c = 15.644 Å, pdf‐No.: 98‐019‐6031). In addition, a second phase with a hexagonal crystal system (also *R3̄̄c*) and lattice parameters a  =  b  =  4.600 Å, c  = 14.763 Å, assigned to pure NiCO_3_ (pdf‐No.: 98‐017‐3985),^[^ ^25^ ^]^ was also identified and is ascribable to the composition of the Ni‐rich rim of the particles.

**Figure 2:**
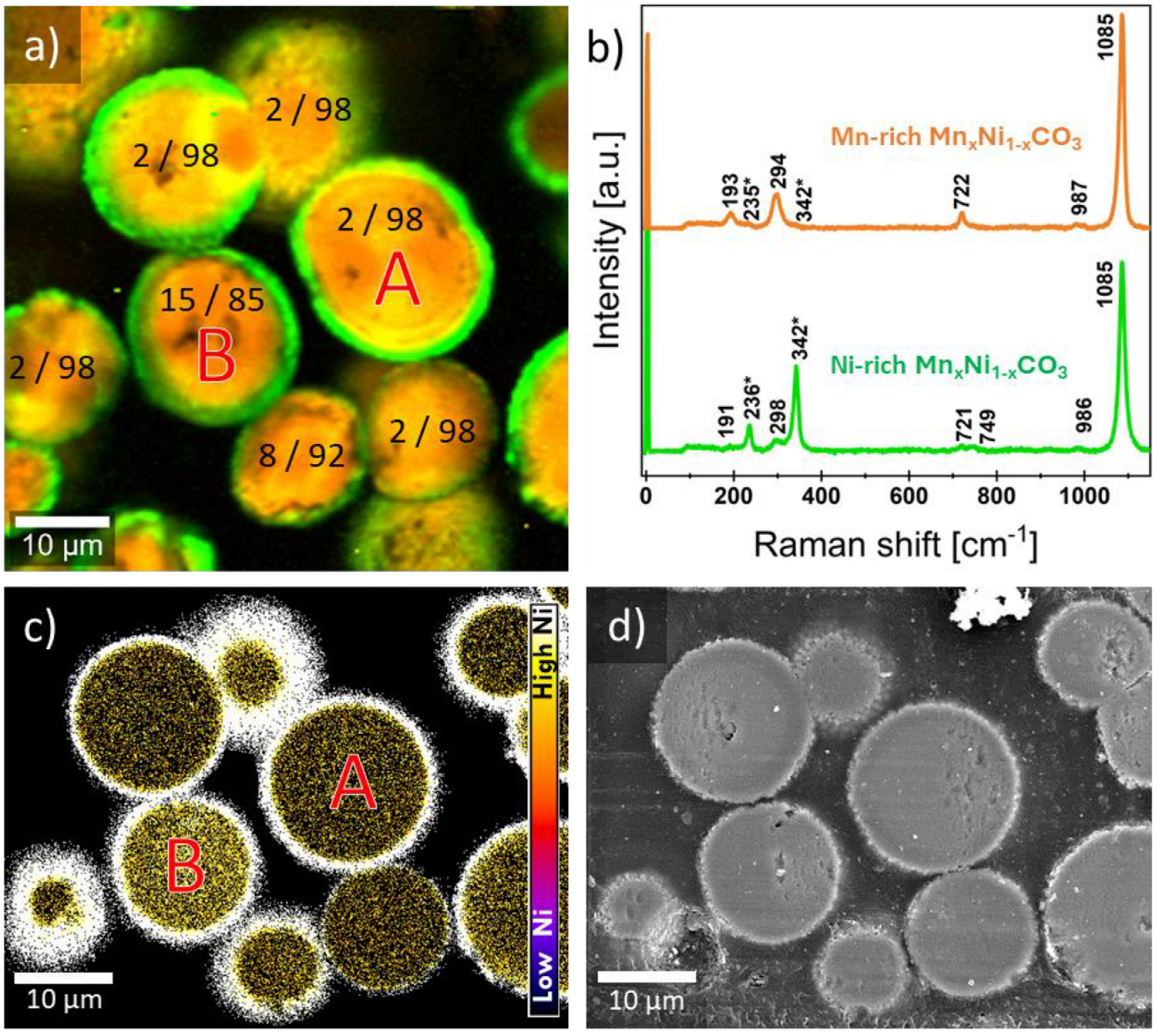
a) Raman imaging of cross‐sectioned precursor particles and b) corresponding cluster Raman spectra for the identified mixed (Mn,Ni)CO_3_ phase (orange) and pure NiCO_3_ phase (green). Blurred image regions indicate signals from particles below the cross‐section plane. Calculated mean Ni/Mn ratios for the particle core composition resulting from Raman measurements are given in a). c) EDX derived image of the Ni/Mn ratio representing the mixed metal distribution over the sample cross‐section shown in a). Note the differing Ni content of particle cores A and B. d) SEM cross section micrograph of the same area confirming the dense packing of primary particles.

Particle cross‐sections were characterized using Raman microscopy and SEM‐EDX analyses. The corresponding results are shown in **Figure** **2**. As the basic structural motif (space group *R3̄̄c*) was identical for the particle rim and the particle interior, similar Raman spectra were obtained irrespective of the position at which the spectrum was measured. However, the increased Mn content for the core regions and the high Ni content of the rim region lead to clear differences in the peaks related to the external E_g_ in the corresponding Raman spectra. The typical Raman modes reported for transition metal carbonates were clearly observed, with a dominant peak at 1085 rel. cm^−1^ assignable to the internal A_1_ _g_ vibrational mode, a minor peak ≈720 rel. cm^−1^ attributable to the internal E_g_ mode, and external E_g_ modes at ≈190 and 300 rel. cm^−1^, respectively.^[^ ^22^, ^26^ ^]^ The low intensity band at 985 rel. cm^−1^ is assignable to the symmetric stretch A_1_ _g_ vibrational mode of the sulphate anion,^[^ ^27^ ^]^ which represents some synthesis educt leftovers. The positions of the external vibrational bands depend on the identity of the transition metal cation in the carbonate. The strong Raman band at 342 rel. cm^−1^ ascribable to external E_g_(L) vibrational mode observed for the particle rim regions corresponds to what is expected for pure NiCO_3_. This band was absent in the spectra measured for the inner particle regions. In addition, the position of the external E_g_ modes at ≈190 and 300 rel. cm^−1^ measured for inner‐particle regions showed clear particle‐to‐particle differences and correlated very well with indicated differences in the qualitative Ni/Mn ratios determined by SEM‐EDX analyses (see Figure 2c). As the position of these modes are known to vary linearly with the Ni/Mn ratio in MnCO_3_/NiCO_3_ exhibiting solid solution behavior,^[^ ^23^ ^]^ the local Ni_x_Mn_1‐x_CO_3_ stoichiometries can be estimated based on the Raman data: The position of the external E_g_ mode for the mixed carbonate is dependent on the amount of Mn and Ni included and shift linearly between the values for pure MnCO_3_ (289 cm^−1^) and pure NiCO_3_ (343 cm^−1^).^[^ ^22^, ^26^ ^]^ Thus, the evaluation of the band position allows to deduce the composition of the mixed carbonate inner‐particle regions. The obtained data suggests that the x‐values in Ni_x_Mn_1‐x_CO_3_ in the in the inner regions of the analyzed particles (“cores”, i.e., excluding the rim region) range between 0.02 ± 0.01 (peak position = 291 ± 1 cm^−1^, particle A) and 0.15 ± 0.01 (peak position = 295 ± 1 cm^−1^, particle A). The obtained mean Ni/Mn‐ratio values for each particle core are given in Figure 2a. A related map showing the weighted peak position assigned to the external E_g_ mode ≈290 cm^−1^ for each pixel can be found in the supporting information (Figure S4, Supporting Information). Finally, the thickness of the rim region consisting of virtually pure NiCO_3_ is estimated to be 1–1.3 µm, and the thickness appears to be identical on all particles.

The results obtained are in line with what is to be expected for the (Mn,Ni)CO_3_ precursor particles prepared according to the synthesis method used, as MnCO_3_ is expected to precipitate first. At intermediate reaction times, when the concentration of Mn^2+^ is already considerably lower, mixed Ni_x_Mn_1‐x_CO_3_ particle cores start to precipitate, where the value of x increases with time. As the Ni^2+^/Mn^2+^ ratio in the solution increases, particle cores with increasing Ni content precipitate from the solution. This leads initially to particles exhibiting virtually pure MnCO_3_ cores, and later to particles having cores with higher Ni/Mn‐ratios up to 15:85. At later stages, with Ni^2+^ still present in solution both due to depletion of MnCO_3_, and the preferential formation of Ni‐ammonia complexes, NiCO_3_ should slowly precipitate out of solution on top of each particle, thus leading to the formation of an almost pure NiCO_3_ rim on the particles, with are relatively uniform thickness of ≈1 µm. Thus, the overall Ni/Mn ratio at the particle level is determined both by the time of precipitation and the particle size, as the NiCO_3_ rim contribution to the overall Ni/Mn ratio of a secondary particle is particle size dependent if the rim layer thickness is constant. So proportionally to volumetric contribution from core and rim, a small particle has a greater Ni contribution originating from the rim compared to a larger particle. Assuming that the particles consists of pure MnCO_3_ cores and pure NiCO_3_ rims with a thickness of 1.2 µm, and considering their respective molar masses and densities, the particles size distribution would render 45% of the total mass Ni‐deficient with respect to the 1/3 Ni/Mn ratio needed for LiNi_0.5_Mn_1.5_O_4_ spinel. Additionally, the total excess Ni present in particles with estimated Ni/Mn ratios exceeding 1/3, and which is expected to be present in secondary Ni‐rich phases after lithiation, is estimated to be ≈6% of the total Ni content.

To conclude, the resulting Ni/Mn ratio for each particle is dependent on two factors: First the size of the particle, as the volume contribution of the Ni‐rich rim sphere decreases with particle size. Second, the time of precipitation and thus the concentration of Ni^2+^ and Mn^2+^ in the solvent during the reaction, as the Mn^2+^ concentration decreases more rapidly compared to Ni^2+^.

### 2.2 Synthesis of o‐LNMO

**Figure 3:**
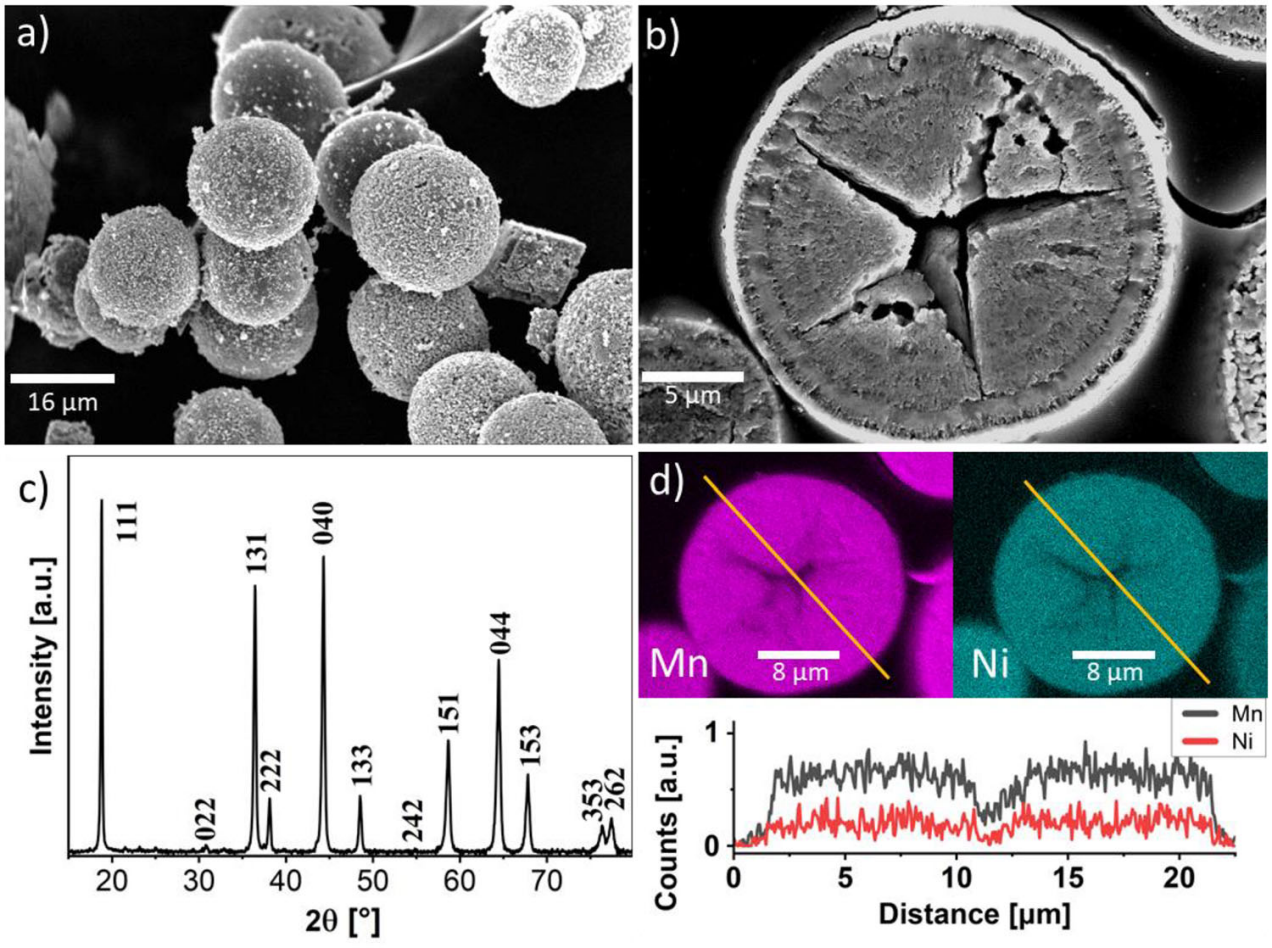
a) SEM images of the final o‐LNMO product after temperature treatment with image‐derived particle size distribution plot (insert) and b) a cross‐section of o‐LNMO particles embedded in EPON. c) The PXRD diffractogram of the o‐LNMO displays reflexes typical for LNMO spinel. d) EDX mapping of the cross‐section shown in b) displaying the distribution of Mn (magenta) and Ni (cyan) within the particles together with an intensity line plot of the two elements across the sample.

The particle size of the precursor was not affected by the thermal treatment, the average particle size and particle size distribution (19 ± 4 µm, n  =  328, particle sizes ranged from 7 to 45 µm, see Figure S3, Supporting Information) obtained for the calcined particles are close to those obtained for the precursor, see Figure 1a and **Figure** **3**a. However, SEM analysis of cross‐sectioned particles revealed an onion‐type structure of concentric rings, and the presence of pores mainly located in the center of the particles, probably as a result of the decomposition of the carbonates (Figure 3b) into oxides and CO_2_. SEM‐EDX analysis of a particle cross‐section showed that the Ni/Mn ratio was constant throughout each particle, highlighting that Ni present as NiCO_3_ in the rim of precursor particles had diffused into and homogeneously distributed itself within the particles. Rietveld analysis (R_wp_ = 8.917, R_p_ = 6.727, Goodness of fit = 2.999) of the XRD pattern shown in Figure 3c) revealed that the material was an almost phase pure spinel (> 99.8%) having a *P4_3_32* symmetry, as expected for ordered LNMO. The value of the lattice parameter a was 8.178 Å, which is in good agreement with the value of 8.170 Å reported for NiCO_3_ (pdf: 98‐007‐0045^[^ ^28^ ^]^). The smaller lattice parameter is reported to result from under stoichiometric amounts of Ni and the subsequent replacement by Mn atoms in the spinel lattice, leading to increased bulk lattice parameters. It corresponds to a stoichiometry of Li_1_Ni_0.46_Mn_1.54_O_4_ in the bulk spinel phase according to a study from Zhong et al.^[^ ^29^ ^]^ A value lower than the determined bulk Ni/Mn ratio of 1/3 in the spinel phase implies the presence of some Ni‐rich impurities, as the bulk chemical composition of Li_0.99±0.05_Ni_0.49±0.02_Mn_1.51±0.05_O_x_ determined via ICP‐MS was very close to the theoretical stoichiometry. Phase analyses suggest the presence of small amounts rock‐salt (< 0.1%) and 0.1% bixbyite impurity phases. The EDX mapping of a randomly selected cross‐sectioned particles shown in Figure 3d reveals a homogeneous distribution of both Mn and Ni. In conclusion, classical bulk characterization reveals that a homogeneous product with minimal impurity phases and evenly distributed transition metals was obtained after thermal treatment of the precursor material.

**Figure 4:**
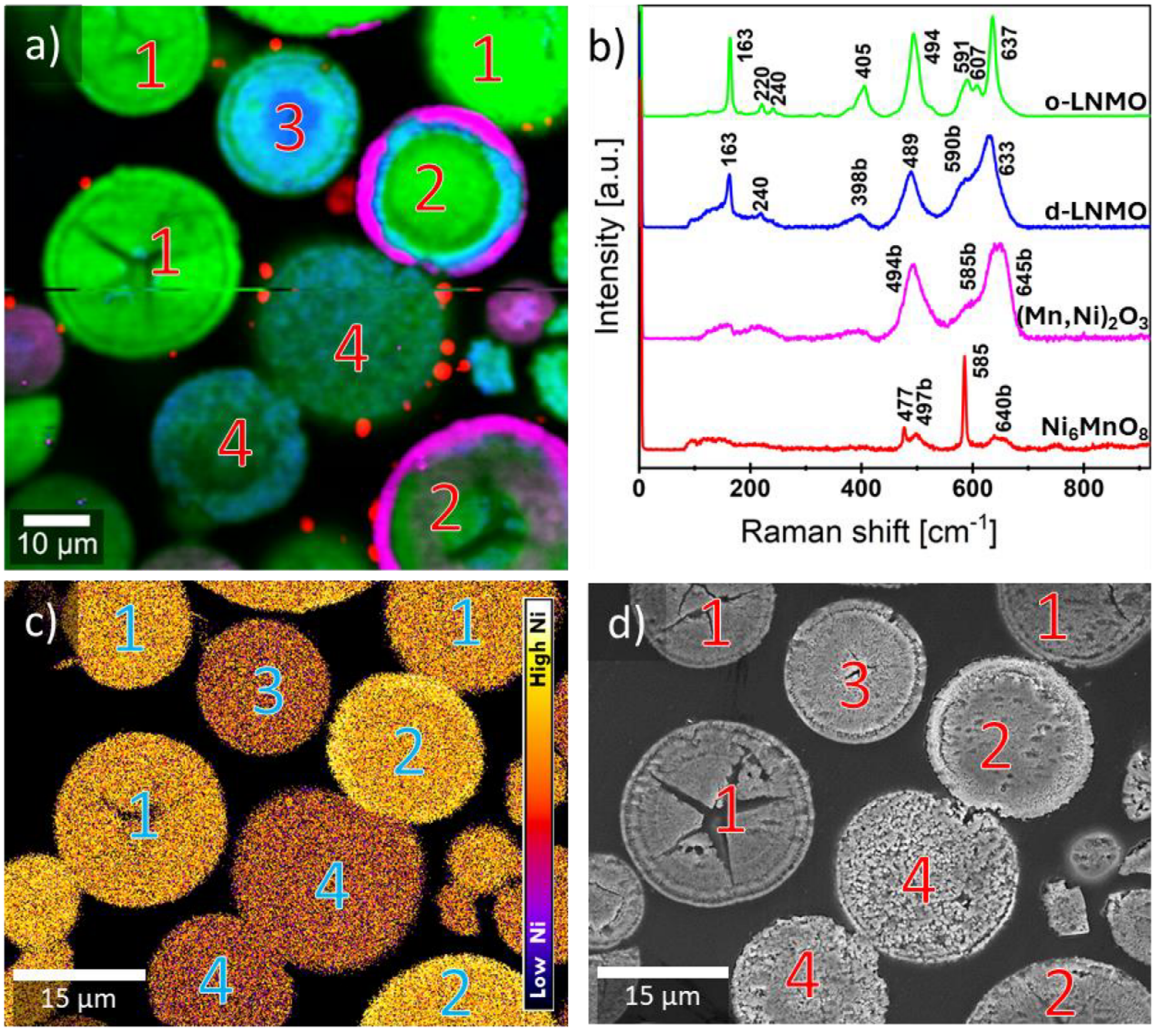
a) Raman imaging of several cross‐sectioned o‐LNMO particles and b) corresponding cluster Raman spectra for the identified ordered (green) and disordered (blue) LNMO phase together with the rock salt (red) and Ni‐rich manganese oxide spinel‐like side phase as impurities (pink). The numbers shown correspond to the particle structure: “1” shows particles consisting solely of homogeneous o‐LNMO, “2” indicates particles with Ni rich side phases located at the rim due to over‐stoichiometric amounts of Ni, “3” corresponds to particles with a disordered LNMO phase in the particle core resulting from less than stoichiometric amounts of Ni, and “4” marks particles with less than stoichiometric amounts of Ni resulting in the d‐LNMO phase randomly distributed. c) EDX derived image of the Ni/Mn ratio representing the mixed metal distribution over the sample cross‐section shown in a): particles marked with “2” show increased Ni content while particles marked with “3” and “4” have a low Ni/Mn ratio. d) SEM cross section micrograph of the same area shown in a) revealing structural heterogeneity of the o‐LNMO material: particles marked with “1‐3″ show an onion‐type structure with dense layers separated by porous intermediate layers, while particles marked with “4” show a sponge‐like, macroporous structure.

To further investigate the homogeneity of the obtained product, multi‐particle characterization was carried out using Raman microscopy in combination with SEM‐EDX analyses over multiple particle cross sections. Ordered LNMO can easily be distinguished from disordered LNMO via Raman spectroscopy due to well‐defined bands located at 163 (F_2g_(1)), 220, and 240 rel. cm^−1^, which are either absent or not well‐separated in Raman spectra of spinel d‐LNMO.^[^ ^30^, ^31^, ^32^, ^33^, ^34^ ^]^ Further, the presence of well‐separated bands in the region from 590–610 cm^−1^, in addition to a strong band in the region 630–650 rel. cm^−1^ assignable to Mn‐O stretching vibrations of MO_6_ octahedra (A_1_ _g_) are evidenced. The Raman spectra measured for particle cross‐sections could be reliably clustered into 4 main spectra shown in **Figure** **4**b, and the location of pixels exhibiting corresponding Raman spectra are shown in Figure 4a using the same color coding.

It is evident that the green spectrum exhibits the characteristics of an ordered spinel LNMO, while the blue spectrum is characteristic for a disordered LNMO. The pink spectrum observed at the rim of some of the particles (marked with “2”) carries resemblance with disordered LNMO, but with additional contributions of another Ni‐rich bixbyite‐type, thus (Mn,Ni)_2_O_3_ phase. The red spectrum can be assigned to a rock‐salt impurity phase, which we, in line with the suggestion of Feltz et al.,^[^ ^35^ ^]^ ascribe to a (probably lithiated) phase isostructural to the Ni_6_MnO_8_ (see Figure S5, Supporting Information for details). This is in good agreement with a recent study by Nisar et al.^[^ ^36^ ^]^ on the nature of secondary phases in LNMO treated at 1000 °C, in which Ni_6_MnO_8_ was identified via coupled Raman spectroscopy and energy dispersive X‐Ray spectroscopy.

Via correlative Raman and EDX analysis, the o‐LNMO particles can be divided in four categories: If the global amounts of Ni and Mn are close the stoichiometric ratio of 1/3 as shown by ICP‐MS elemental analysis, Ni distributes evenly throughout the whole particle and leads to a homogenous o‐LNMO phase (particles marked with “1”). If the Ni/Mn ratio is above 1/3 (indicating over‐stoichiometric amounts of Ni, e.g. particles marked with “2”), Ni infiltrates throughout the particle until the stoichiometric composition of Ni_0.5_Mn_1.5_ is uniformly reached everywhere, leading to the formation the o‐LNMO phase (green spectrum). The excess Ni remains in the rim region of the particle, which leads to the formation of the disordered Ni‐rich (Mn,Ni)_2_O_3_ phase, isostructural to a bixbyite phase (pink spectrum), as mentioned above. In particles with less than stoichiometric amounts of Ni, an additional, probably slightly Ni‐deficient d‐LNMO phase (blue spectrum) is formed as the lattice sites of the missing Ni are taken by Mn atoms. This happens either in the center of the particle (particles marked with “3”) or randomly distributed (particles marked with “4”). The expected cause for this behavior is likely related to the inner morphology of the respective particles, which is discussed below.

The results of the correlative EDX analysis for the obtained cross‐sectioned o‐LNMO particles are shown in Figure 4c,d. Particles exhibiting a high Ni content (Figure 4c), particles labelled “1” and “2”) appear in a yellow hue consist mainly of ordered spinel LNMO, as confirmed by Raman microscopy analysis (Figure 4a). This yellow color represents a Ni/Mn‐ratio close to 1/3, as o‐LNMO only forms when the ratio is close to the stoichiometric values. Particles appearing brown in the SEM‐EDX maps (Figure 4c), particles labelled “3” and “4”), i.e., having a lower Ni/Mn ratio than those appearing yellow, show the presence of disordered LNMO in the Raman microscopy images. Thus, the absence of the o‐LNMO phase to different extents in these particles can be ascribed to an overall Ni/Mn ratio lower than 1/3. Interestingly, it appears that the inner particle structure is dependent on the Ni/Mn ratio, where near stoichiometric ratios correspond to a compact structure and lower than stoichiometric ratios to more porous, “sponge”‐like structures. Furthermore, the rim regions on some particles consisting of disordered LNMO appear to show a higher Ni/Mn ratio than for regions consisting of the ordered phase, in good agreement with the Raman results (Ni‐rich spinel phase at rim).

So, in conclusion, although a near perfect bulk Ni/Mn ratio was achieved, there are differences in phase and elemental compositions on the particle level. In order to explain these results, which are closely linked to the effective Ni/Mn ratio on the o‐LNMO particle level, it is important to recall the structure of the precursor particles. Slight particle‐to‐particle variations in the Ni/Mn ratio were observed in the cross‐sectional SEM‐EDX analysis of the precursor particles. This particle‐to‐particle variation agrees with the particle size‐dependent variation in the Ni/Mn ratio of the precursor particles discussed above, where about half of the LMNO mass is expected to be Ni‐deficient, and ≈6% of the Ni should be contained in Ni‐rich impurity phases mainly associated with particles exhibiting a Ni/Mn ratio > 1/3.

### 2.3 Synthesis of d‐LNMO

As in the case of the o‐LNMO, no substantial change in particle size (19 µm ± 4 µm, n  = 354, particle sizes ranged from 8 to 38 µm, see Figure S6, Supporting Information) occurred upon lithiation and thermal treatment using the temperature program leading to the d‐LNMO modification. PXRD Phase analysis suggested that the material was virtually phase pure (> 99%) LNMO spinel with the expected *Fd3̄̄m* symmetry and a lattice parameter of 8.205 Å, which is in good agreement which the value of a  =  8.210 Å reported for typical disordered LNMO phase (pdf: 98‐018‐8989^[^ ^37^ ^]^). Extrapolation from the unit cell dimensions reported by Zhong et al.^[^ ^29^ ^]^ results in an average stoichiometry of Li_1.00_Ni_0.33_Mn_1.67_O_4_ in the bulk LNMO spinel phase. A low Ni‐content in the spinel phase can be understood, as Ni partly separates from the spinel phase and forms thermodynamically favored Ni‐rich oxides under the high temperatures applied. Although these oxides are less thermodynamically stable at lower temperatures, the Ni cannot re‐enter the spinel phase due to the rapid cooling, as suggested by Feltz et al. In addition to the spinel phase, a rock salt impurity phase was identified in the diffractogram (as shown in the Figure S6c, Supporting Information, <1%).

**Figure 5:**
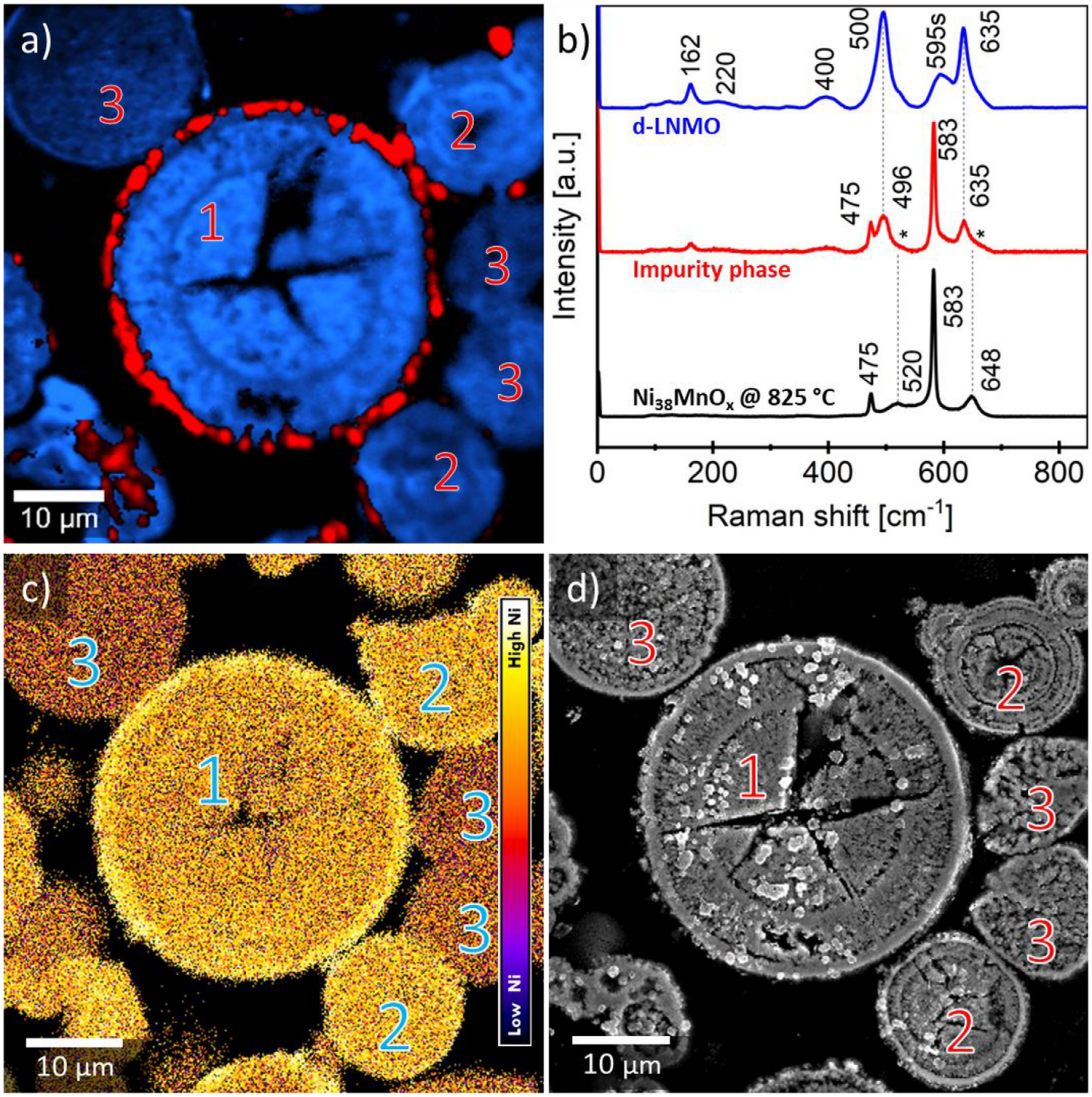
a) Raman imaging of cross‐sectioned d‐LNMO particles and b) corresponding cluster Raman spectra for the observed disordered phase (blue), together with the rock salt phase (red) secondary phase. The black spectrum is related to the synthesized artificial Ni_38_MnO_x_ rock salt impurity phase. c) EDX derived image of the Ni/Mn ratio representing the mixed metal distribution over the sample cross‐section shown in a): particles marked with “1” show a relatively high Ni/Mn ratio (close to 1/3) and an additional Ni rich rim, particles marked with “2” show the same core Ni/Mn ratio as particle “1”, but without a Ni‐rich rim. Particles marked with “3” inhibit a comparably lower Ni/Mn ratio throughout the particle. d) SEM cross section micrograph of the same sample area shown in a) reveal an internal structure similar to the o‐LNMO material, “1” and “2” indicating an onion‐like structure and “3” a sponge like structure.

Raman microscopy (**Figure** **5**a,c) confirmed that the d‐LNMO particles consisted of disordered LNMO. As the Raman signature of disordered LNMO spinel shows broader peaks and overall less distinct features compared to that of ordered LNMO, the influence of the particle level Ni/Mn ratios on the formation of disordered LNMO could not be verified. However, particle‐particle differences in the Ni/Mn ratio could be evidenced by SEM‐EDX mapping, as shown in Figure 5c. Furthermore, comparison of EDX maps with SEM images (Figure 5b,d) suggested morphological differences (compact versus porous) between particles depending on their Ni/Mn ratio, where particles that exhibited a Ni/Mn ratio close to 1/3 showed a much more compact structure. As shown in Figure 5b and in more detail in Figure S5 (Supporting Information), the Raman spectrum for the particle impurity phase can be described as an overlay of the Raman spectrum of Ni_7.6_Mn_0.2_O_8_ with the spectrum observed for the d‐LNMO bulk phase, again suggesting the presence of a Ni‐rich impurity phase (Ni_6_MnO_8_ defect rock salt‐type, Fm3m^[^ ^38^, ^39^ ^]^) at the rim of the particles which initially exhibited a high overall Ni/Mn ratio. Thus, also in the case of these d‐LNMO particles, the inhomogeneous Ni/Mn distribution within the (Mn,Ni)CO_3_ precursor particles has important implications for their compositional and structural characteristics, as under‐stoichiometric amounts of Ni lead to the formation of d‐LNMO with increased amounts of Mn(III), and over‐stoichiometric amounts of Ni lead to the formation of Ni rich impurities, especially in the particle shell.

### 2.4 Post‐Transformation of d‐LNMO into o‐LNMO

The possibility of transforming the d‐LNMO into o‐LNMO was investigated by applying the temperature program used for o‐LNMO modification to the d‐LNMO material. Such particles will be referred to as d‐o‐LNMO particles in the following. Again, the additional temperature treatment did not affect the particle size distribution nor the particle morphology (17 µm ± 5 µm, n  = 660, particle sizes ranged from 6 to 42 µm, see Figure S9, Supporting Information). The PXRD pattern revealed the typical reflections expected for ordered LNMO with a lattice parameter a  =  8.175 Å, corresponding to a stoichiometric Ni coefficient of ≈0.46.^[^ ^29^ ^]^ Keeping in mind that the d‐LNMO particles used had an Ni stoichiometric coefficient of only 0.33, a re‐distribution of Ni from impurity phases present in the disordered particles into the spinel phase has occurred during tempering. Rietveld analysis suggested that the material consisted of ≈99.9% LNMO spinel, with minor impurities in the form of 0.1% bixbyite and trace amounts of the rock salt phase. However, with a Ni‐stoichiometric coefficient in d/o‐LNMO of 0.46, slightly under the desired Ni/Mn‐ratio of 1/3, the amount of impurity phases based on this analysis is most probably underestimated.

**Figure 6:**
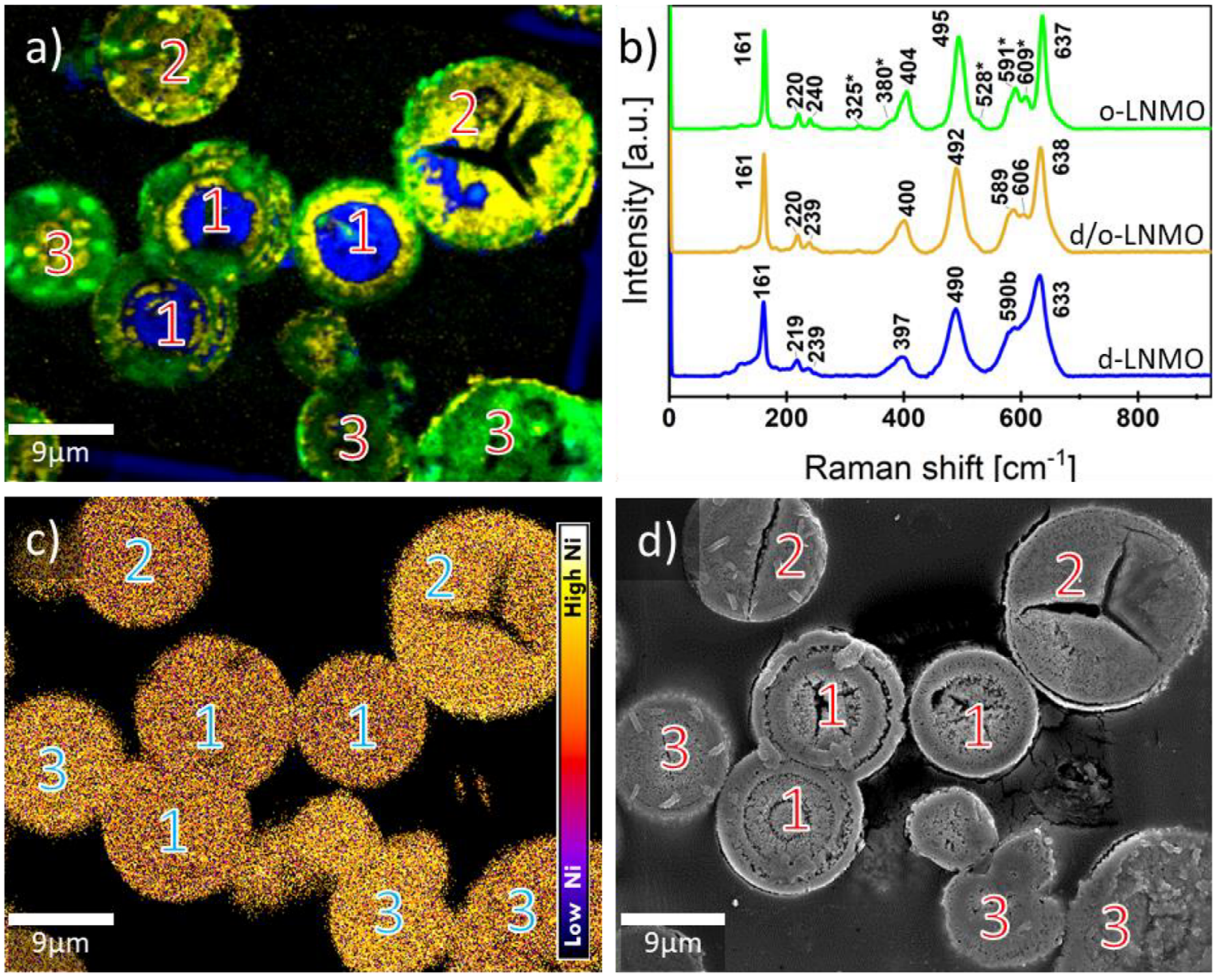
a) Raman imaging of cross‐sectioned d‐o‐LNMO particles and b) corresponding cluster average Raman spectra for the observed ordered (green), mixed ordered and disordered (yellow) and disordered (blue) LNMO phase. The numbers indicate the phase composition of particles: “1” indicates particles with a o‐LNMO phase at the rim and a d‐LNMO phase in the core, with a “semi‐ordered” intermediate phase between them. “2” indicates particles showing the intermediate phase throughout the whole particles, whereas particles marked with “3” show mostly a homogenous o‐LNMO phase. c) Ni/Mn ratio derived from EDX: particles marked with “3” inhibit a higher Ni/Mn ratio compared to particles marked with “1” or “2” d) SEM micrograph of the same d‐o‐LNMO sample area shown in a). The compositional heterogeneity from the precursor level and the structural intricacies of the o‐ and d‐LNMO samples are conserved through the second temperature treatment, particles marked with “1” show thick (up to 8 µm), porous areas especially at the core. Particles marked with “2” and “3”, in contrast, are compact, showing only major cracks, but not the porous structure seen in particles marked with “1”.

The EDX maps of the particle sections show a homogeneous internal distribution of Ni and Mn with a constant ratio per particle, as shown in **Figure** **6**c. However, as for the other samples, a clear particle‐particle variation in the Mn/Ni ratio could be observed. When looking at the internal structure of the d‐o‐LNMO particles, the morphology can be described as a mix of porous, sponge‐like structure in the center, surrounded by compact, onion‐like layers of material, as seen in the SEM images in Figure 6d. Notably, some particles (Figure 6 “1”) exhibited a core‐shell structure, with ordered spinel in the outer shell and a disordered spinel core (green and blue) in Figure 6a,b. The contact area of both phases was characterized by a transition‐phase with mixed d‐ and o‐LNMO features, which can be seen by the lower intensity of the split Raman bands ≈590–610 rel. cm^−1^. It should be noted that, similar to the o‐LNMO sample, where particles with a Mn/Ni ratio below 1/3 showed patches of less ordered or even disordered LNMO, the ordered shell of this sample also exhibited patches with cation disorder. The ordered phase exclusively formed in the compact region of the onion‐like structure, while the disordered phase remained in the porous, sponge‐like region. As mentioned above, the formation of the disordered phase involves a phase separation process of the spinel above a critical temperature of ≈650 °C,^[^ ^35^ ^]^ where a Ni‐rich phase (rock salt like) starts to separate and forms around the spinel crystallites. This shifts the Ni/Mn‐ratio in the spinel off the optimal stoichiometry for the highly ordered spinel, causing cation disorder. It seems that the reversal of this phase separation is easier within the compact areas of the material, while the mobility of ions in the porous phase could be structurally hindered due to limited contact between the individual crystallites, as to why the phase separation cannot reverse and the material remains in less ordered state. Thus, although there were particle‐particle variations in the overall Ni/Mn‐ratios, the most important influence on the re‐occurrence of ordering in the spinel phase appears to be the influence of local porosity on Ni diffusion during tempering.

### 2.5 Electrochemical Analysis

In addition, the Ni distribution in the spinel phase was characterized by electrochemical measurements. First, a Ni/Mn ratio in the spinel phase lower than 1/3leads to the occupation of the Ni sites with Mn(III) atoms, which results in an additional Mn(III)/Mn(IV) transition peak ≈4,1 V versus Li/Li^+^. Second, the degree of cation ordering in the spinel phase leads a potential shift, resulting in different voltage gaps between the Ni(II)/Ni(III) and Ni(III)/Ni(IV) transition peak potentials.^[^ ^40^ ^]^

Charge‐discharge curves for the three different LNMO materials were recorded performing tests using type 2032 coin cells with metallic lithium as counter electrode, in LP30 electrolyte. From these charge–discharge curves, subsequently d*Q*/d*U* plots were calculated. From the charge‐discharge measurements, the specific capacity was evaluated, which was found to be ≈120 mAh g^−1^ for all three synthetized LNMO materials. This is relatively close to the theoretical capacity of 147 mAh g^−1^,^[^ ^41^ ^]^ taking the determined impurities into account. Rate capability and cycling stability were tested for each material, and showed expected LNMO behavior. The results are found in the supporting information (Figures S10 and S11, Supporting Information). Interestingly, all materials perform well at high C‐rates, which can be attributed to a relatively high porosity and thus a higher surface area. The d*Q*/d*U* plots yield information about the relative contributions of ordered and disordered domains in the LNMO materials as well as about their stoichiometry. First, if the Ni/Mn ratio in the spinel phase is lower than 1/3, which was observed for all three LNMO materials given the results of the PXRD analyses, some of the Mn is present as Mn^3+^, giving rise to an oxidative peak in the d*Q*/d*U* plots at ≈4.1 V versus Li/Li^+^, reflecting the oxidation of Mn(III) to Mn(IV). Thus, the overall Ni and Mn stoichiometric factors can be evaluated by integration of the Mn(III)/Mn(IV) transition peak ≈4,1 V versus Li/Li^+^ and the Ni(II)/Ni(III) and Ni(III)/Ni(IV) transition peaks ≈4.7 V versus Li/Li^+^. Second, as discussed in detail by Song et al.,^[^ ^40^ ^]^ the cation ordering has an impact on the voltage gap between the Ni(II)/Ni(III) and Ni(III)/Ni(IV) transition peaks ≈4.7 V versus Li/Li^+^: It is larger for d‐LNMO (≈ 55 mV) as compared to o‐LNMO (≈20 mV), and an increased separation between these peak potentials for o‐LNMO is also observed if the Ni/Mn ratio is lower than 1/3.^[^ ^42^ ^]^ These values are in good agreement with the voltage gaps measured for our samples (15 mV for o‐LNMO and 54 mV for d‐LNMO). A recent study by Stüble et al.^[^ ^43^ ^]^ revealed that for ordered, stoichiometric LNMO, the peak separation is ≈19 mV, ≈45 mV for an ordered LNMO with a Ni‐stoichiometric coefficient of 0.43, and 58 mV for disordered LNMOs having stoichiometric coefficients of 0.44 and 0.48, respectively.

**Figure 7:**
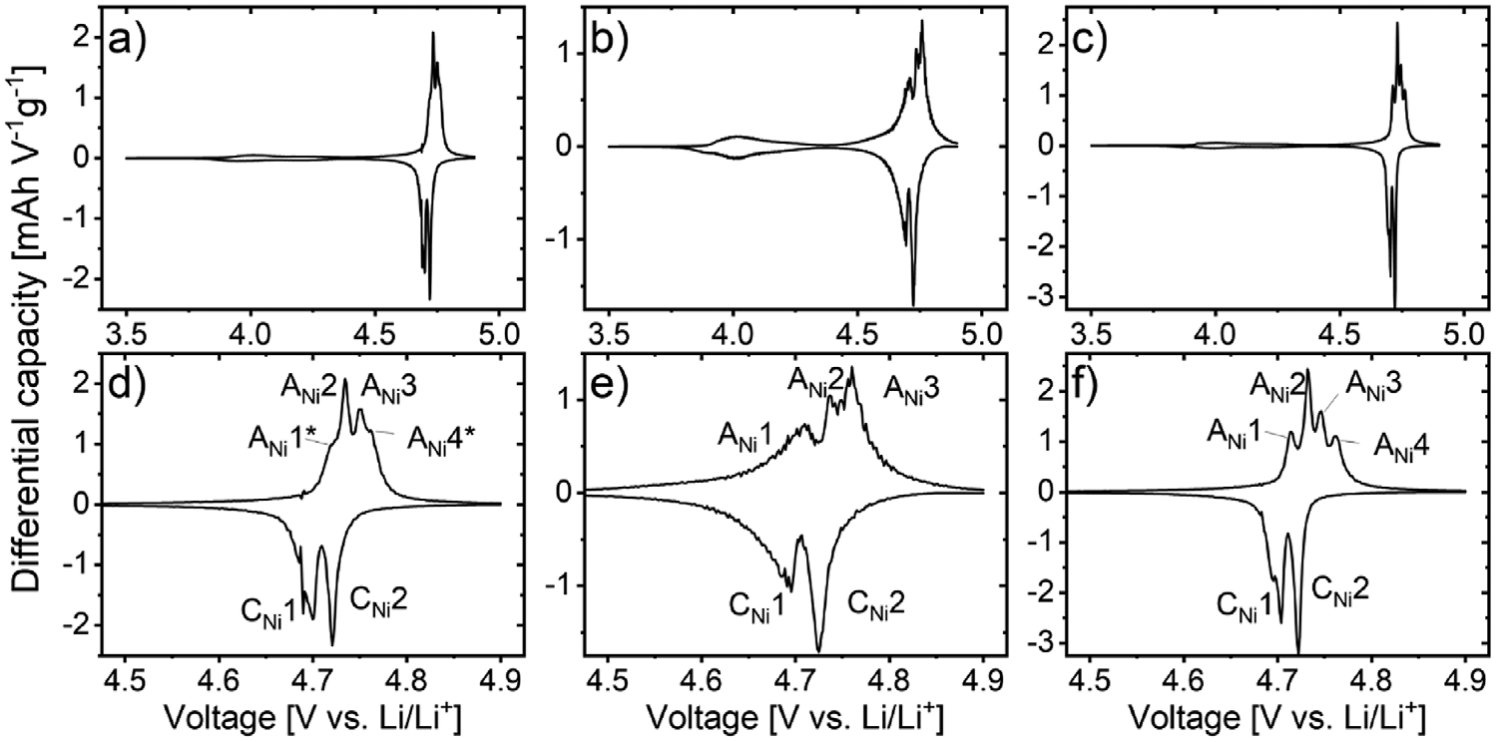
dQ/dU‐plots calculated from the second cycle of charge‐discharge curves measured at c/10. Using the a) o‐LNMO, b) d‐LNMO and c) d‐o‐LNMO material. The full curves a–c) for each sample show the Mn(III)/Mn(IV) transition in the region of 3.8–4.4 V versus Li/Li^+^ and the peaks associated with Ni(II)/Ni(III) and Ni(III)/Ni(IV) transitions in the region ≈4.5–4.9 V versus Li/Li^+^. Zoom‐in dQ/dU‐plots d–f) show the Ni‐associated potential region from 4.5–4.9 V versus Li/Li^+^, which reveal characteristic differences in peak separation and intensity shifts associated with the material composition.

The calculated d*Q*/d*U*‐plots derived from the charge/discharge results are shown in **Figure** **7**a–c in the potential region of 3.5–5.0 V versus Li/Li^+^, where the prominent redox peaks are located. In good agreement with the estimated stoichiometries based on PXRD, a higher (≈18%) contribution of the Mn(III)‐to‐Mn(IV) transition is seen for d‐LNMO as compared to o‐LNMO and d‐o‐LNMO (both ≈10%). Based on the relative contribution of the Mn(III)/Mn(IV) transition to the total capacity of each investigated material, the stoichiometry of the materials can be estimated. For the o‐LMNO and d‐o‐LNMO materials, Ni‐stoichiometries of 0.42 were obtained, in fair agreement with the stoichiometries estimated based on the PXRD analyses, which was 0.46 in both cases. For d‐LMNO, the electrochemical analysis results a Ni stoichiometry of 0.37, which is slightly higher than the 0.33 derived from the PXRD‐based analysis. Thus, although there are some deviations between values, both methods provide values that match quite well for the o‐LNMO and the d‐o‐LNMO bulk phase, which have comparable Ni/Mn values and are slightly Ni‐deficient, while the “disordered” LNMO bulk phase is highly Ni‐deficient.

The clearest influence of the material inhomogeneities on the electrochemical performance of the different LNMO materials was seen for the Ni‐oxidation peaks occurring between 4.5–4.9 V versus Li/Li^+^, which is why zooms of this region from the d*Q*/d*U*‐plots are also shown in Figure 7d–f. The d*Q*/d*U* plot calculated for the o‐LNMO sample shows 4 peaks, two narrow peaks located at 4.737 and 4.752 V versus *Li/Li^+^*, and two broader shoulder peaks at ≈4.719 and 4.763 V versus *Li/Li^+^*. We assign these narrow “inner” peaks to the Ni(II)/Ni(III) and Ni(III)/Ni(IV) transitions of stoichiometric, ordered LNMO based on their peak separation of 15 mV, as well as their narrow peak shape. The broader peaks at 4.719 and 4.763 V versus *Li/Li^+^* are correspondingly assigned to Ni(II)/Ni(III) and Ni(III)/Ni(IV) transitions of non‐stoichiometric, ordered LNMO due to the peak separation of 44 mV. The broadening of these peaks is assumed to originate from the presence of non‐stoichiometric LNMO particles with different Ni/Mn‐ratios. The small spacing between the inner and outer peaks pairs and uncertainty about the individual peak shape makes quantification of the different contributions through peak‐fitting difficult, but the contribution of stoichiometric o‐LNMO seems to be higher than that of non‐stoichiometric o‐LNMO.

The Ni‐region of the d*Q*/d*U*‐curve for d‐LNMO material (Figure 7b) is clearly less well‐defined compared to o‐LNMO, with a strong tailing toward low potential values, and three local maxima located at 4.710, 4.743, and 4.764 V versus *Li/Li^+^* respectively, where the contribution of the middle peak is clearly the smallest. The voltage gap between the two main peak potentials is 54 mV, i.e., very close to the 58 mV, which has been observed for disordered LNMO by Casas‐Cabanas et al.^[^ ^42^ ^]^ However, the lower‐voltage region appears as a not very well defined, broad peak ≈4.70 V versus *Li/Li^+^*. Also, the peak at 4.743 V assigned to a Ni(III)/Ni(IV) transition could have a “sister” Ni(II)/Ni(III) peak in that region with a similar peak separation. Comparing the corresponding results obtained for o‐LNMO, we assign the two broad Ni(III)/Ni(IV) peaks observed for d‐LMNO to particles with a broad range of Ni/Mn ratios, where the peak positioned at higher voltage values originates from particles with lower Ni/Mn ratios than those giving rise to the peak at 4.743 V versus *Li/Li^+^*.

The most well‐defined d*Q*/d*U*‐curve was obtained for d‐o‐LNMO. This may not be surprising, as this material was tempered for the longest time of the three studied samples, possibly increasing the crystallinity of d‐o‐LNMO, as well as the cation ordering, due to a prolonged time in the temperature window in which the cations are mobile. For this material, also 4 clearly separated peaks were observed, positioned at 4.715 V, 4.732 V, 4.747 V, and 4.762 V versus *Li/Li^+^*, i.e., virtually identical positions as the corresponding peaks for the o‐LNMO material. Thus, this data gives clear evidence for a successful disordered to ordered LNMO transition in the d‐o‐LNMO material. However, the compositional differences in Ni/Mn ratio between different particles lead to the formation of both stoichiometric and Ni‐deficient o‐LNMO particles. As discussed above, an 50% of the LNMO should be Ni‐deficient as estimated based on the precursor particle size distribution. This is in good agreement with the percentage of charge associated with the A_Ni_1+4 transition peaks relative to the total charge associated with the Ni transitions, which was determined to be 47% for the d‐o‐LNMO modification and 46% for the o‐LNMO modification. Overall, the electrochemical results are therefore in excellent agreement with the SEM‐EDX and Raman microscopy results. They also suggest that the mean‐values of the stoichiometric coefficients that can be derived from PXRD analyses are some weighted averages of a distribution of stoichiometries at the particle‐to‐particle level.

## 3 Conclusions

Microparticles of LNMO were synthesized through co‐precipitation of Ni‐ and MnCO_3_ followed by thermal lithiation and subsequent thermal treatment. Via a correlative approach using space resolved Raman‐ EDX‐ and SEM analysis applied on the same cross sectioned particles, detailed information on the particle composition was revealed on the precursor and functional material level. In a typical batch coprecipitation process using a mixture of urea and DMC as carbonate sources, spherical (Mn,Ni)CO_3_ precursor particles with a mean diameter of ≈ 19 ± 4 µm were obtained. As carbonate formation is a result of the self‐supporting decomposition of urea and DMC in the solution under hydrothermal conditions, a gradual increase in the carbonate concentration takes place. Therefore, almost pure MnCO_3_ particle cores precipitated during the early stages of the synthesis due to the lower solubility product, while particle cores precipitating at a later stage show increasing Ni content, according to the metal ion concentration in the solution at the time of precipitation. After depletion of Mn in the solution, the remaining Ni^2+^ precipitated as pure NiCO_3_ shell at the end of the synthesis, leading to the formation of particles with a MnCO_3_ enriched core and a NiCO_3_ shell around the core. This led to particle‐to‐particle variations in the Ni/Mn ratios, where both the composition of the solution at the time of precipitation and the secondary particle size contributed to this effect. It was shown that the individual composition of particles, especially regarding the Ni/Mn ratio, has a direct influence on the stoichiometry of the LNMO phase formed after lithiation, and subsequently on the formation of secondary phases. Most notably the distribution of Ni is of great importance, as the Ni content plays a crucial role defining the electrochemical characteristics of the LNMO phase. This in turn had a direct influence on the electrochemical performance of the synthesized materials as active cathode material for Li‐ion battery as demonstrated via cycling in half‐cells versus Li/Li^+^ and subsequent calculation of d*Q*/d*U* plots. Additionally, it was found that the formation of undesired secondary phases is directly connected to the transition metal distribution. While it is known that the formation of secondary phases, like Ni‐rich rock salt, takes place und typical conditions during LNMO synthesis, our correlative approach allows the localization of these secondary phases in the particles and is also capable of giving significant insights into their chemical and structural composition. Differences in solubility products between different metal hydroxides or carbonates used in the synthesis of MMO may to a certain extent be balanced through a rational choice or complexing agents. However, local differences in the chemical composition of primary particles are not easy to counter‐balance, although the length‐scale at which such inhomogeneities occur will be system‐dependent. Chemical inhomogeneities can partly be eliminated through tempering, but if they, as in our case, exist on a particle‐to‐particle level, these will most likely also remain after thermal treatment.

**Figure 8:**
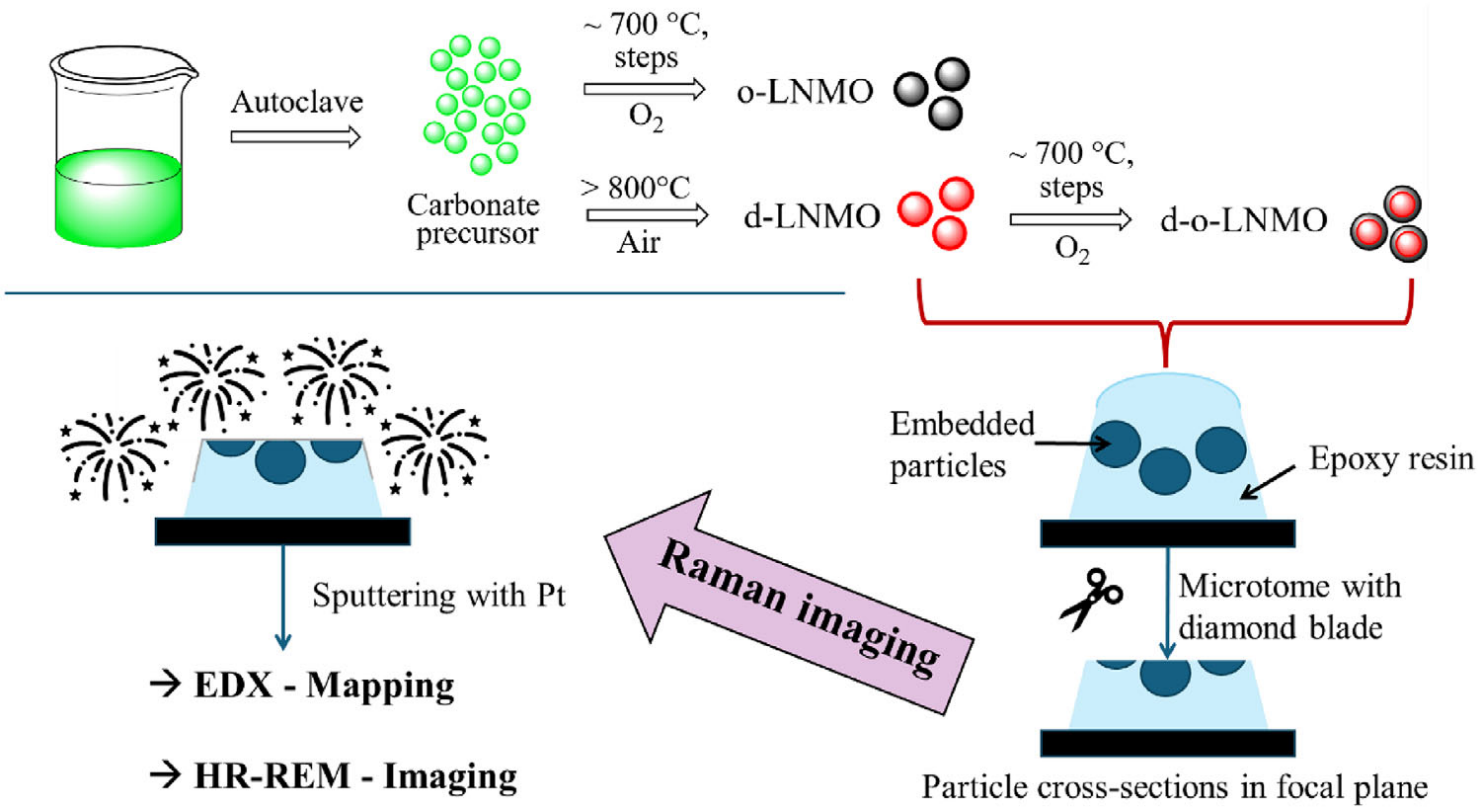
Schematic illustration depicting the precursor synthesis, thermal treatment routes and workflow of subsequent sample preparation and characterization of identical particle cross‐sections via correlative Raman/SEM/EDX‐Analysis.

## 4 Experimental Section

### Synthesis

*Mixed Carbonate Precursors*: For the hydrothermal synthesis of the spherical, mixed carbonate precursors, a modified synthesis based on the work of Liu et al.,^[^ ^13^ ^]^ MnSO_4_·H_2_O (4.75 g, 28.1 mmol, VWR Chemical AnalaR Normpur), NiSO_4_·6 H_2_O (2.53 g, 9.63 mmol, Sigma–Aldrich, Reagent Plus), Urea (7.98 g, 132.9 mmol, Merck, pro analysis) and dimethyl carbonate (5.10 g, 56.6 mmol, Sigma–Aldrich, for synthesis) were dissolved in deionized water (150 mL). Also, cetyltrimethylammonium chloride (CTAC, 0.3 mL of a 25% solution) in water was added (Sigma‐Aldrich). The resulting solution was transferred to a 200 mL PTFE‐lined autoclave and the sealed reactor was heated to 200 °C for 5 h in a rotary furnace (90 Kh^−1^, 5 rpm during heating). After cooling to room temperature, the resulting light green product was filtrated and washed with a 1:1 mixture of EtOH and deionized water to remove the soluble (NH_4_)_2_SO_4_ side product and unreacted educts. The washed powder was dried at 60 °C for 24 h.

### LNMO Spinel Products

Different lithiation agents including LiNO_3_, LiOH, and Li_2_CO_3_ were evaluated. Superior outcomes were attained with Li_2_CO_3_; whereas alternative reagents led to increased impurity levels, either from non‐lithiated precursor remnants (namely Mn_2_O_3_ and MnNiO_3_) or elevated rock‐salt modifications (see XRD diffractograms in S2).

For the synthesis of the ordered LNMO‐modification (Sample “o‐LNMO”), in a first lithiation step (**L**), the (Mn,Ni)CO_3_ precursors were lithiated with Li_2_CO_3_ (Fluka chemica, puriss. p.a., Li/metals  =  1.05:1) in a muffle oven (B 150, Nabertherm) under air atmosphere at 725 °C for 12 h, heating rate: 140 K h^−1^. Afterwards the product was cooled down to RT. In the next step **1**), the lithiated particles were heated with a rate of 145 K h^−1^ to 760 °C in a tube furnace (RHTH 120–150/18, Nabertherm) under oxygen atmosphere, cooled down (−130 K h^−1^) to 500 °C **2**) and then heated (130 K h^−1^) to 760 °C again **3**), with the temperature held for 2 h for steps **1–4**) shown in **Table** **1**. This activation was described by Aktekin et al.^[^ ^44^ ^]^ to avoid the introduction of oxygen deficiencies, impurities, and crystal defects. Next, the temperature was lowered to 710 °C **4**). This temperature was chosen as it is below the oxygen release temperature (>725 °C), but still above the temperature window in within the cation ordering‐disordering transition occurs (<680 °C). In the final heating step **5**), the sample was slowly cooled down (−40 K h^−1^) to 310 °C over a period of 10 h to enable cation ordering to occur. After this, the sample was cooled down naturally to RT **6**). For the disordered LNMO‐modification (“d‐LNMO”), the precursor material was mixed with LiCO_3_ and then heated to 825 °C in a muffle oven under air with a heating rate of 165 K h^−1^. After holding the temperature for 12 h, the furnace was deactivated, and the product naturally cooled down to RT **7**). For the product resulting from the post‐transformation of the d‐LNMO into the o‐LNMO modification (“d‐o‐LNMO”), the d‐LNMO particles resulting from the synthesis above was treated with the same program used for the ordered LNMO modification under oxygen atmosphere.

**Table 1:** Temperature programs utilized for the synthesis spinel modifications.

Sample	Starting material	Thermal treatment	Atmosphere
Calcined & lithiated LNMO	(Mn,Ni)CO_3_‐precursors	(**L**) 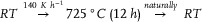	Air
Ordered LNMO^[^ ^44^ ^]^	Lithiated LNMO particles	(**1**) 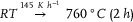 (**2**) 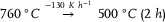 (**3**) 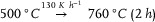 (**4**) 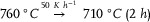 (**5**) 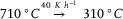 (**6**) 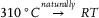	Oxygen
Disordered LNMO	(Mn,Ni)CO_3_‐precursors	(**7**) 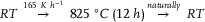	Air
Core‐shell LNMO	d‐LNMO particles	Identical program as used for the ordered LNMO product	Oxygen

### Synthesis of Ni_6_MnO_8_, Ni_18_MnO_x_ and Ni_38_MnO_x_ Rock Salt “Impurity Compounds”

The synthesis of the hexa‐nickel manganese oxide was achieved via a route proposed by Porta et al.,^[^ ^45^ ^]^ utilizing the “oxalate precursors”‐technique starting from the respective Mn(II)‐ and Ni(II)‐ acetates (Thermo Fisher Scientific, 99+% and Alfa Aesar, 99+%). To this end, the acetates were dissolved in 25 % acetic acid and mixed in the desired stoichiometric ratios of Mn:Ni, ranging from 1:38 to 6:1 without the addition of Li. This mixture was heated to 100 °C under reflux and stirred continuously, then a solution of oxalic acid (Merck, 99+%, stoichiometric amount + excess 10 % in 20 mL of demin. water) was quickly added. After the resulting mixed oxalate precipitated, the precursor product was dried in air at 105 °C. The resulting powder was calcinated in two batches under air atmosphere either at 600 °C or at 825 °C for 12 h each (heating rate 160 K h^−1^).

### Material Characterization

PXRD measurements were performed in reflection mode on an X‐Ray diffractometer (X'Pert Pro, Malvern Panalytical) with Cu K_α_ radiation to determine the crystal structure of the materials. The crystal structure was identified and refined with the Rietveld method using the “HighScore Plus” software by Panalytical. The particle morphology and size distribution were examined by scanning electron microscopy (SEM, Hitachi S‐5200 Kryo) operated with an acceleration Voltage of 10 kV. For the SEM‐imaging the LNMO powders were fixed on adhesive carbon tape. The manual particle size evaluation was performed with the software ZEN 2.6 (blue edition, ZEISS). Particle cross‐sections used for correlative energy dispersive X‐Ray (EDX)‐ and confocal Raman‐Imaging were obtained by embedding the particles in epoxy resin (Science Services, EPON EMbed‐812 Kit: EMbed‐812, NMA, DDSA DMP‐30 activator) and subsequent cutting via microtome (Leica, model Reichert Ultracut S with a Diatome type Trim 45 trimming knife). The EDX mappings and HR SEM images for the LNMO particle cross sections were performed with a Quanta 3D FEG FIB‐SEM microscope (Thermo Fisher Scientific), utilizing an element specific detector (EDAX Ametek) and the APEX standard 3.0 software package (both EDAX Ametek). Multiple scans were performed (MnK and NiK peaks, 15 kV, 8 nA, 200 µs dwell time, 512 × 400 px, 32 frames) and the measured counts accumulated for Mn and Ni, respectively. The evaluation of the EDX mappings was performed with ImageJ software.^[^ ^46^ ^]^ The absolute accumulated Mn (MnK) and Ni (NiK) counts were evaluated for each pixel and then divided accordingly to get Ni/Mn ratios. The resulting Ni/Mn‐ratios were normalized for all materials measured to enable a comparison of all ratios observed and are represented by the same scale bar. As the calculated ratios do not represent the real stoichiometry due to factors as background noise, they should be seen as a semi‐quantitative indicator for the Ni and Mn distribution. Using this method, a relative comparison between areas with differing Ni/Mn‐ratios is possible.

The Raman‐measurements were performed on a confocal Raman microscope (WITec alpha300 R) utilizing a YAG‐laser (WITec, λ = 532 nm, 1.0 mW laser power) and a VIS spectrometer (WITec UHTS 300) with a spectral resolution of ± 1 rel. cm^−1^. Raman images of particle cross sections were collected by scanning the cross‐sectioned surface with an 50x ZEISS objective with a numerical aperture (N_A_) of 0.7, leading to an approximate lateral resolution of 450 nm or 2 pixels µm^−1^ as derived from the objective N_A_ and the excitation wavelength via the *Rayleigh‐*criterion. The datasets were analyzed using the software Project 5.0 from WITec. Background correction was performed for each recorded spectrum using a shape filter (size: 200 pixels, circular shape) and the intense peaks caused by cosmic radiation were removed. The resulting dataset was first subjected to a cluster analysis, which groups areas with similar spectra together via the K‐means approach. From these clusters, areas composed of overlapping spectra were determined using demixing tools provided by the Project 5.0 software and the spectra of the individual pure components were calculated. By combining the Raman spectra of the individual components and the imaging dataset through linear combination of the individual spectra, the phase composition of the sample was approximated for each pixel. The cluster affiliation is represented by a designated color; a mixed phase is represented by additive color mixing. The overall spectrum intensity is shown by the brightness of each pixel. Quantitative elemental analysis for Li, Mn, and Ni was performed via inductively coupled plasma mass spectrometry (ICP‐MS, Thermo Fisher Scientific, iCap Q).

### Electrochemical Measurements

Electrochemical testing was performed on type 2032‐coin cells with metallic lithium as counter‐electrode (450 µm thick) and two layers of glass fiber sheets (Whatman GF/A) as separator. The cathodes were prepared with a composition of 94 % active material, 3 % polyvinylidene fluoride (PVDF, Solvay, Solef 51 310) and 3 % carbon (Super P, Timcal) as conductor in N‐methyl‐2‐pyrrolidone (NMP, Sigma–Aldrich, battery grade). The slurry was coated (active material loading: 9–10 mg cm^−2^, layer thickness: 150 µm) onto an aluminum foil current collector and dried for 12 h at 80 °C. Calendaring was performed twice with a pressure of 20 N/mm^2^ at 100 °C. The electrolyte used was a commercially purchased LP 30 electrolyte (battery grade, Sigma Aldrich), consisting of a 1 M LiPF_6_ solution in a 1:1 mixture (v/v %) of dimethyl carbonate (DMC) and ethylene carbonate (EC). The cell was assembled in a glovebox (MBraun Labmaster) under an argon atmosphere (99.99 %) with H_2_OF and O_2_ content below 0.1 ppm. The electrochemical experiments were performed on a BaSyTec CTS‐LAB battery cycler. Rate capability was tested with c‐rates of C/10, C/5, C/3, C/2, 1C, 2C, 3C, 5C and 10C (five cycles each) at RT. Cycling stability was also tested, with an additional 100 cycles with a C‐rate of 1C. d*Q*/d*U* plots were calculated for the second cycle at a c‐rate of C/10. A schematic summary of the workfow is given in Figure 8.

## Conflict of Interest

The authors declare no conflict of interest.

## Supporting information



Supporting Information

## Data Availability

The data that support the findings of this study are available from the corresponding author upon reasonable request.
