# Supplementary material for: Precursor Inhomogeneities Influence the Properties of Multimetal Oxides as Shown for LiNi0.5Mn1.5O4 Derived from Hydrothermally Synthesized Precursors
Source: Adv Sci (Weinh). 2025 Jul 27;12(36):e08174. doi: 10.1002/advs.202508174 (PMC12462943; doi:10.1002/advs.202508174)
Supplement: Supplementary file 1 — Supporting Information [file ADVS-12-e08174-s001.docx]

**Supporting information**

**Precursor Inhomogeneities Influence the Properties of Multimetal Oxides as Shown for LiNi_0.5_Mn_1.5_O_4_ Derived from Hydrothermally Synthesized Precursors**

Simon Schauer,^1^ Bastian Beitzinger,^1^ Gregor Neusser,^2^ Christine Kranz,^2^ Mika Lindén^1,*^

**S1: SEM image - Size distribution for (Mn,Ni)CO_3_ precursor particles**

**
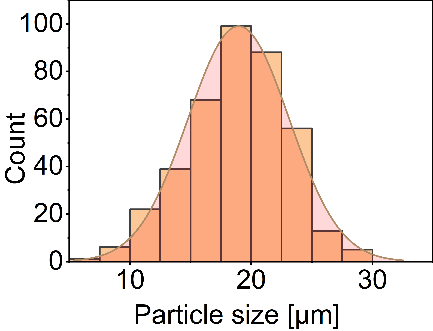

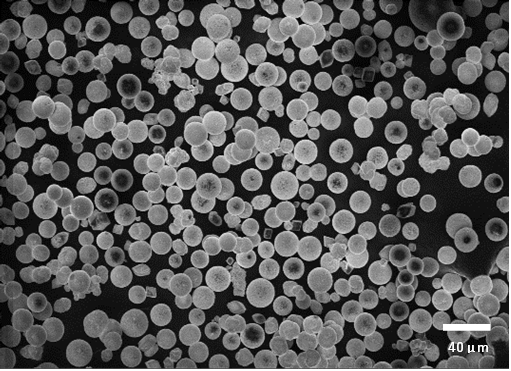
**

Figure S1: *SEM image of the synthetized (Mn,Ni)CO_3_ precursor particles, recorded for the size evaluation. The size evaluation was done by image analysis using the ZEN 2.6 software from ZEISS.*

**Table ST1: Size distribution for (Mn,Ni)CO_3_ precursor particles (n=398)**

| Data | Diameter [µm] |
| --- | --- |
| Average | 18,96 |
| Std. dev. | 4,18 |
| Minimum | 7,20 |
| Median | 19,10 |
| Maximum | 44,94 |

**Table ST2: Elemental analysis for the (Mn,Ni)CO_3_ precursor via ICP-MS (n=3)**

| Element | w-% | Std. dev. [w-%] | Stoichiometry | Std. dev. |
| --- | --- | --- | --- | --- |
| Mn | 33,99 | 0,02 | 1,50 | 0,01 |
| Ni | 12,12 | 0,01 | 0,50 | 0,01 |
| Ni/Mn ratio | - | - | 0,333 | - |

**S1.1: Raw NiK and MnK EDX images for the (Mn,Ni)CO_3_ precursor**

**
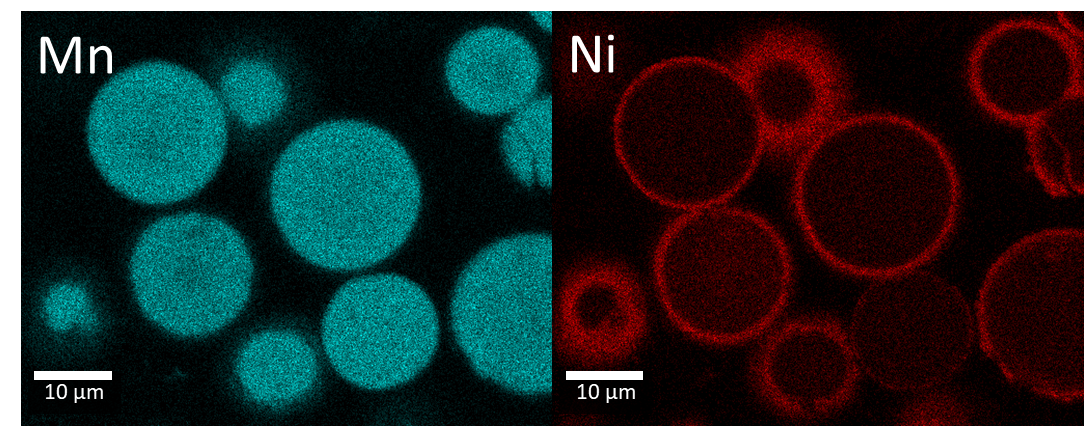
**

Figure S1.1: *Images showing the raw data recorded for the MnK and NiK channels via EDX analysis applies to the (Mn,Ni)CO_3_ precursor. These were used to calculate the images showing the Ni/Mn-ratio in Fig. 2 c) by division of the NiK through the MnK channels.*

**S2: PXRD results for the (Mn,Ni)CO_3_ precursor lithiation with Li_2_ CO_3_, LiOH and LiNO_3_ at 700 °C:**

**
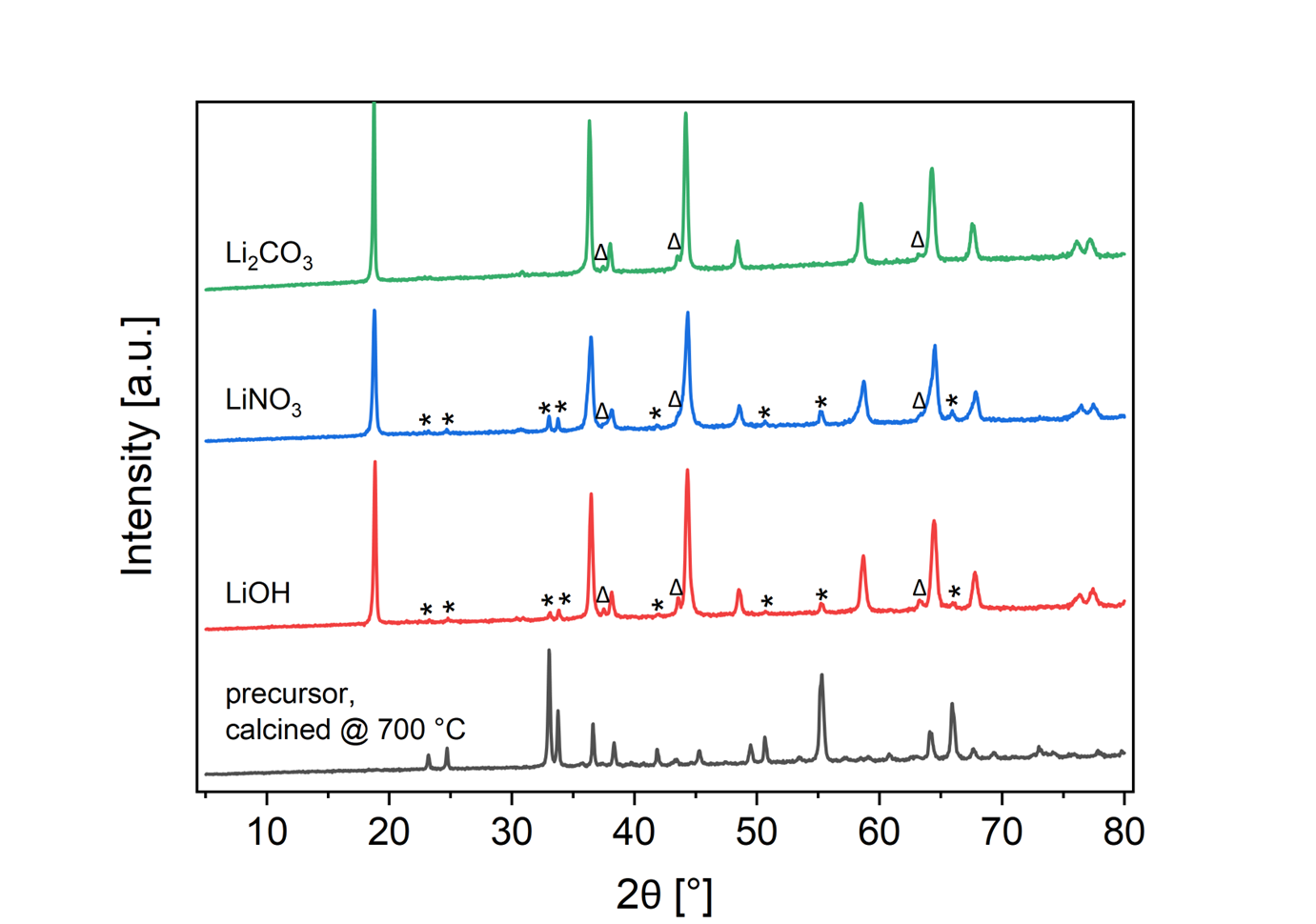
**

Figure S2: *PXRD diffraction patterns results for the pre-calcined (without Li) (Mn,Ni)CO_3_ precursor (black) and LNMO spinel synthesized with different lithium sources, namely Li_2_CO_3_ (green), LiNO_3_ (blue) and LiOH (red). The pre-calcination of the precursors without Li results in a roughly 50:50 mixture of Mn_2_O_3_ and MnNiO_3_. For the lithiated products, the reflections corresponding to oxidized precursor leftovers are marked with an asterisk (*). The reflections associated with the rock-salt phase are indicated with a triangle (Δ). Refinement parameters: pre-calcined precursors - R_wp_ = 10.859, R_p_ = 7.684, Goodness of fit = 3.046; Lithiated with LiCO_3_ (ca. 3 % rock salt): R_wp_ = 11.555, R_p_ = 6.955, Goodness of fit = 4.195; Lithiated with LiNO_3_ (ca. 2 % of each Mn_2_O_3_ and rock salt, ca. 4 % MnNiO_3_): R_wp_ = 9.353, R_p_ = 7.047, Goodness of fit = 2.961; Lithiated with LiOH (ca. 3 % rock salt, 2 % MnNiO_3_ and 1 % Mn_2_O_3_) R_wp_ = 7.610, R_p_ = 5.600, Goodness of fit = 3.404.*

**S3: SEM image: Size distribution for ordered LNMO product particles**

**
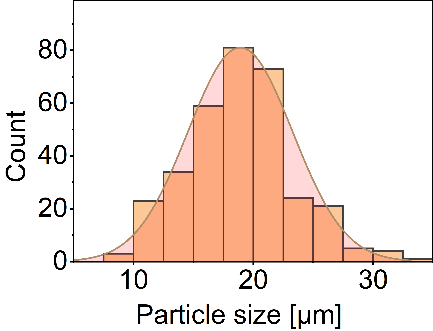

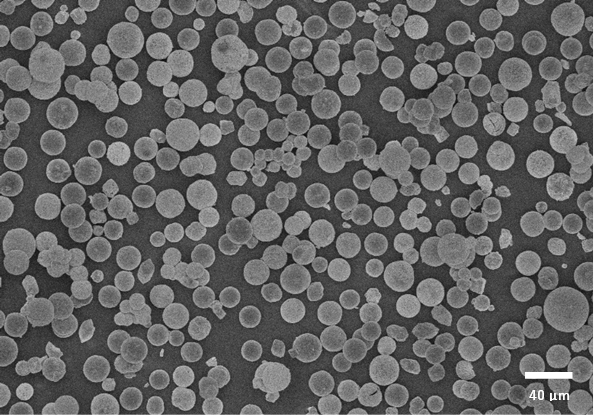
**

Figure S3: *SEM image of the spinel particles synthesized via the thermal treatment route for the ordered LNMO modification.*

**Table ST3: Size distribution for ordered LNMO product particles (n=328)**

| Data | Diameter [µm] |
| --- | --- |
| Average | 18,90 |
| Std. dev. | 4,37 |
| Minimum | 8,53 |
| Median | 18,91 |
| Maximum | 33,25 |

**Table ST4: Elemental analysis for ordered LNMO product via ICP-MS (n=3)**

| Element | w-% | Std. dev. [w-%] | Stoichiometry | Std. dev. |
| --- | --- | --- | --- | --- |
| Mn | 34,10 | 0,62 | 1,52 | 0,07 |
| Ni | 12,20 | 0,41 | 0,51 | 0,02 |
| Li | 3,84 | 0,09 | 0,97 | 0,05 |

**S3.1: Raw NiK and MnK EDX images for the o-LNMO product**

**
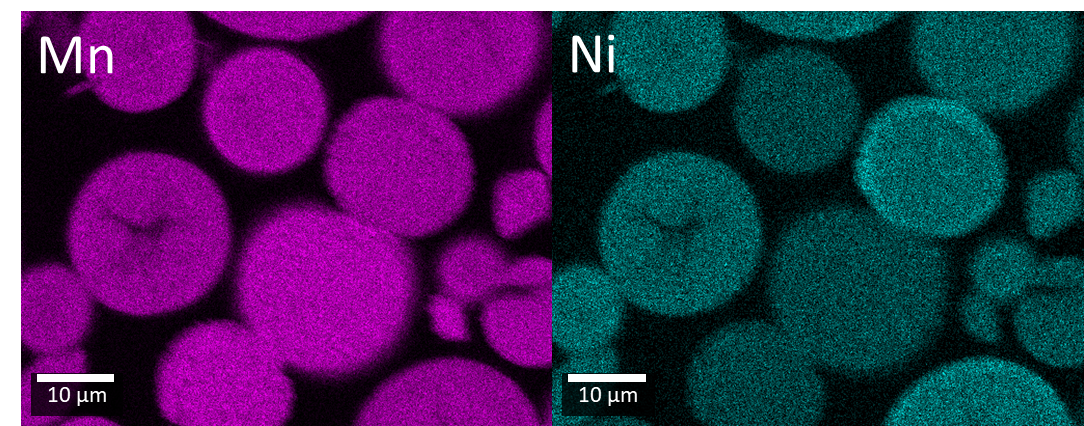
**

Figure S3.1: *Images showing the raw data recorded for the MnK and NiK channels via EDX analysis applied to the o-LNMO product. These were used to calculate the images showing the Ni/Mn-ratio in Fig. 4c) by division of the NiK through the MnK channels.*

**S4: Raman map of the external E_g_-mode peak position around 295 rel. cm^-1^**


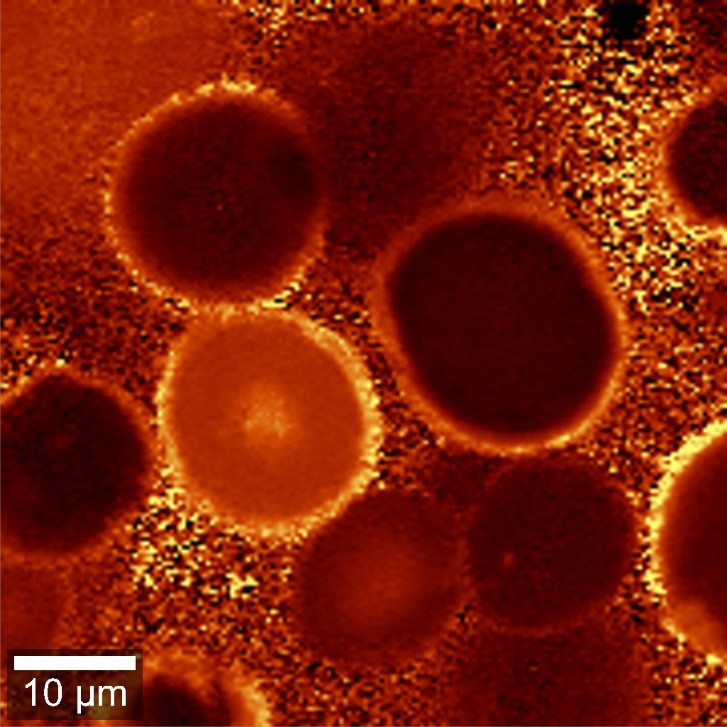

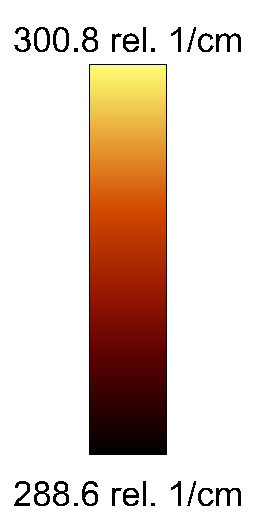


Figure S4: *Raman map of the weighted peak position of the E_g_-mode for the mixed (Mn,Ni)CO_3_ precursor particle cross sections cores for each pixel. The position of this peak shifts between 289 rel. cm^-1^ for pure MnCO_3_ and 343 rel. cm^-1^ for pure NiCO_3_^22,29^. The resulting peak position linearly correlates with the amounts of Ni and Mn in the mixed carbonate, showing solid solution behavior*^3^*. Please note that this is only applicably to the core areas of the particle cross sections, as the Ni-rich rim area shows a uniform peak position around 342 rel. cm^-1^, related to an almost pure NiCO_3_ layer around every particle.*

**S5: Raman spectra obtained for the artificial impurity phases derived from Ni_x_Mn_y_C_2_O_4_-precursors.**

**
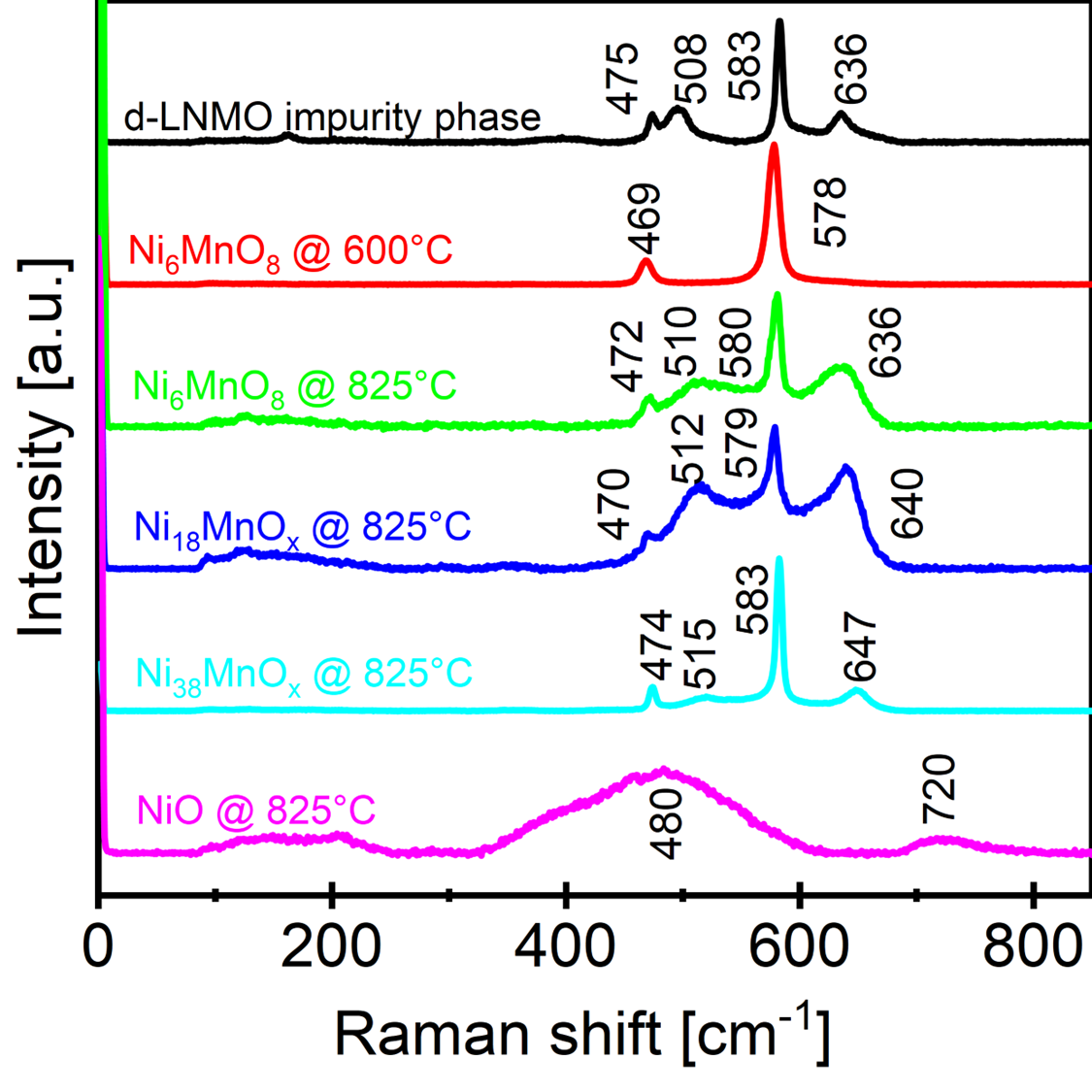
**

Figure S5: *Raman spectra obtained for the artificial impurity compounds derived from calcination of the Ni_x_Mn_y_C_2_O_4_-precursors at the temperatures given.*

**S6: SEM, size distribution, cross-section SEM, PXRD and single particle EDX for disordered LNMO spinel product particles**

**
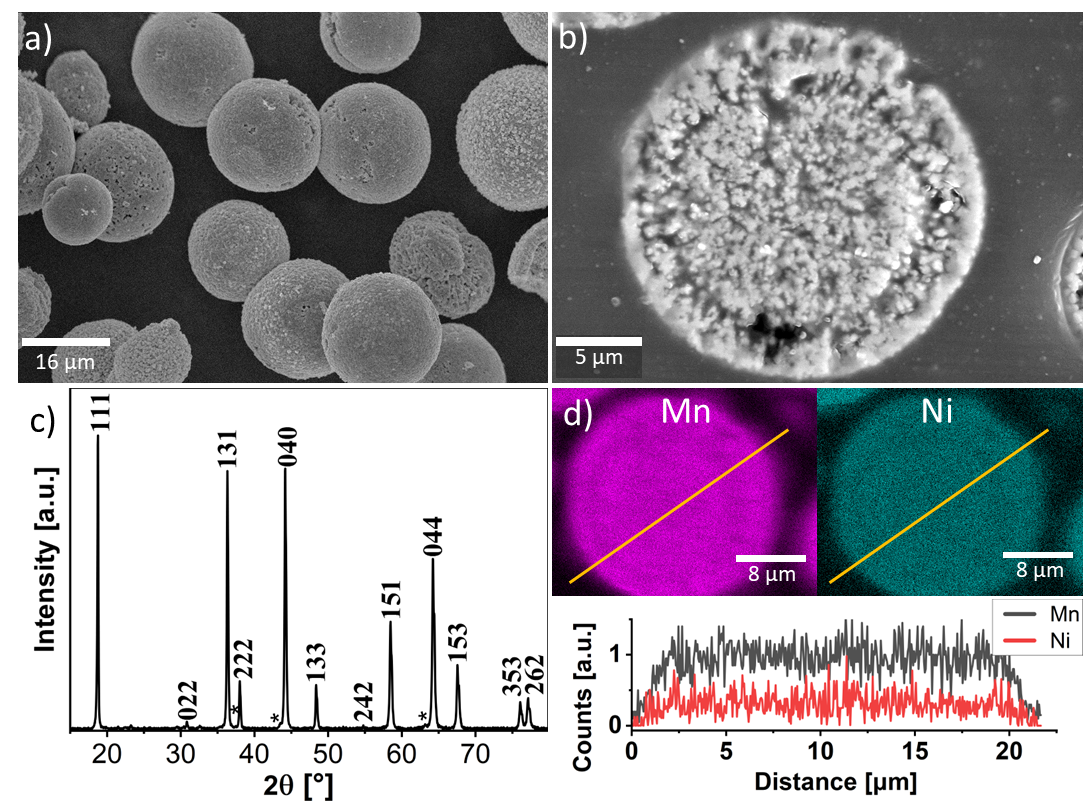
**

Figure S6: *a) SEM image of the final d-LNMO product particles with the size distribution measured for the batch in the inlay in the top right corner, b) a SEM image of a particle cross section from the same batch, c) the obtained powder diffractogram with typical reflexes for LNMO-spinel and the impurity phase identified as defect rock salt (Ni_6_MnO_8_ structure type, marked with *, R_wp_ = 7.165, R_p_ = 4.792, Goodness of fit = 2.740) and d) the EDX imaging for Mn and Ni of the same particle cross section shown in b) (color code: Mn = magenta, Ni= cyan). At the bottom, the line plot obtained from the EDX imaging along the line indicated in orange is shown.*

**S7: SEM image size distribution for disordered LNMO product particles**

**
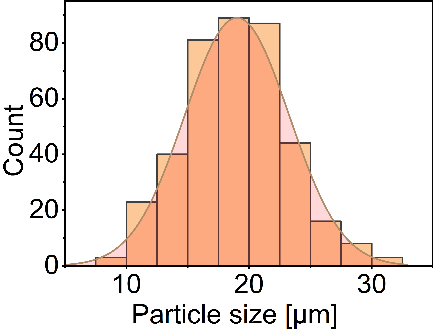

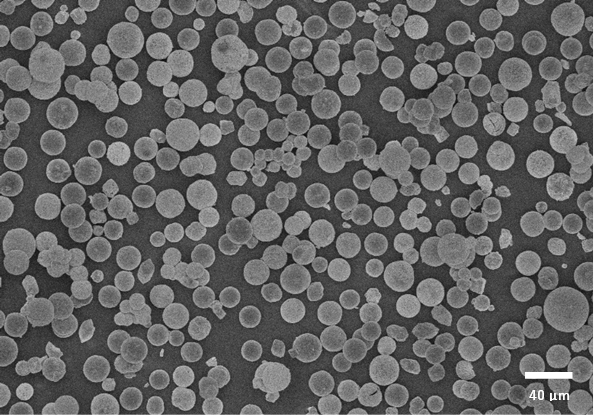
**

Figure S7: *SEM image of the spinel particles synthetized via the thermal treatment route for the disordered LNMO modification, recorded for the size evaluation.*

**Table ST5: Size distribution for the disordered LNMO product particles (n=354)**

| Data | Diameter [µm] |
| --- | --- |
| Average | 19,01 |
| Std. dev. | 4,26 |
| Minimum | 8,16 |
| Median | 19,19 |
| Maximum | 38,20 |

**Table ST6: Elemental analysis for the disordered LNMO product via ICP-MS (n=3)**

| Element | w-% | Std. dev. [w-%] | Stoichiometry | Std. dev. |
| --- | --- | --- | --- | --- |
| Mn | 41,75 | 2,12 | 1,48 | 0,14 |
| Ni | 15,66 | 0,19 | 0,52 | 0,03 |
| Li | 3,59 | 0,15 | 1,01 | 0,08 |

**S7.1: Raw NiK and MnK EDX images for the d-LNMO product**

**
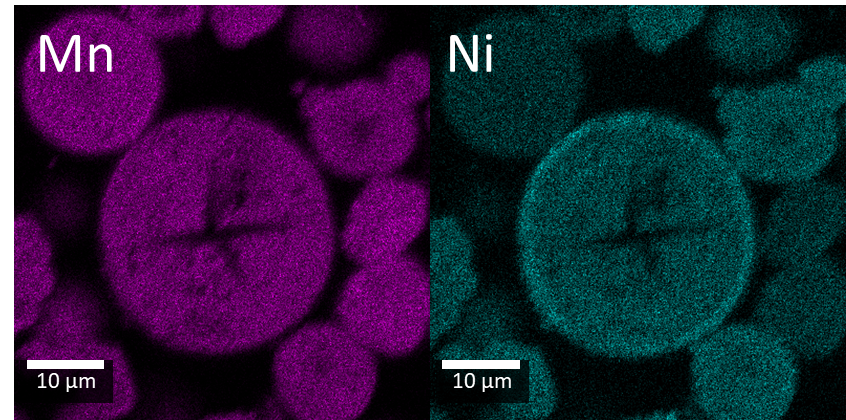
**

Figure S7.1: *Images showing the raw data recorded for the MnK and NiK channels via EDX analysis applied to the d-LNMO product. These were used to calculate the images showing the Ni/Mn-ratio in Fig. 5c) by division of the NiK through the MnK channels.*

**S8: SEM, size distribution, cross-section SEM, PXRD and single particle EDX analysis of the d-o-LNMO spinel product**

**
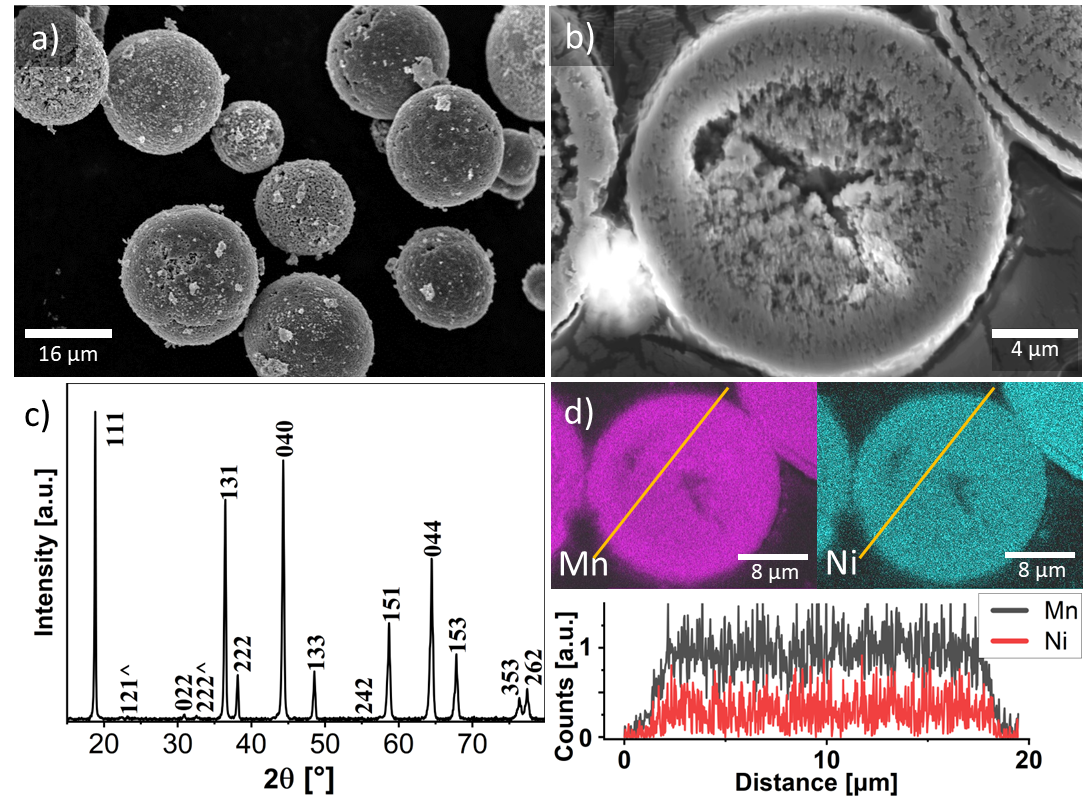
**

Figure S8: *a) the SEM image the final core-shell LNMO product particles with the particle size distribution of the batch in the inlay in the top right corner, b) a SEM image of a particle cross section from the same batch, c) the obtained powder diffractogram with typical reflexes for LNMO-spinel and bixbyite remnants (marked with ^, R_wp_ = 9.643, R_p_ = 6.931, Goodness of fit = 3.279) and d) the EDX imaging for Mn and Ni of the same particle cross section shown in b) (color code: Mn = magenta, Ni= cyan). At the bottom, the line plot obtained from the EDX imaging along the line indicated in orange is shown.*

**S9: SEM image: Size distribution for d-o transformed LNMO product particles**

**
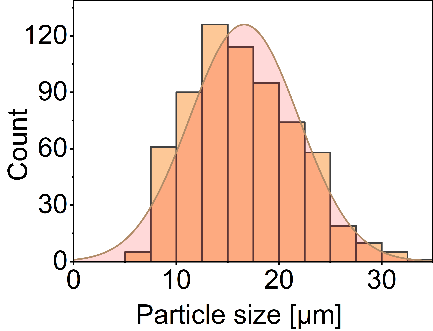

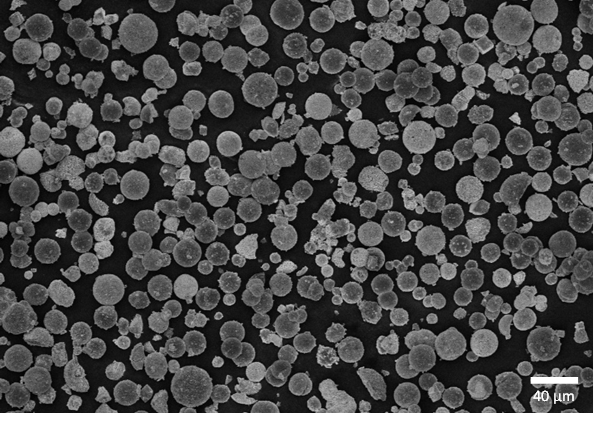
**

Figure S9: *SEM image of the spinel particles synthetized via the thermal treatment route for the disordered LNMO modification with subsequent treatment for the ordered LNMO modification applied, recorded for the size evaluation.*

**Table ST7: Size distribution for the d-o transformed LNMO product particles (n=660)**

| Data | Diameter [µm] |
| --- | --- |
| Average | 16,61 |
| Std. dev. | 5,31 |
| Minimum | 5,28 |
| Median | 16,15 |
| Maximum | 41,59 |

**Table ST8: Elemental analysis for the d-o transformed LNMO product via ICP-MS (n=3)**

| Element | w-% | Std. dev. [w-%] | Stoichiometry | Std. dev. |
| --- | --- | --- | --- | --- |
| Mn | 46,30 | 0,92 | 1,51 | 0,05 |
| Ni | 16,59 | 0,35 | 0,51 | 0,02 |
| Li | 3,85 | 0,10 | 0,99 | 0,05 |

**S9.1: Raw NiK and MnK EDX images for the d-o-LNMO product**

**
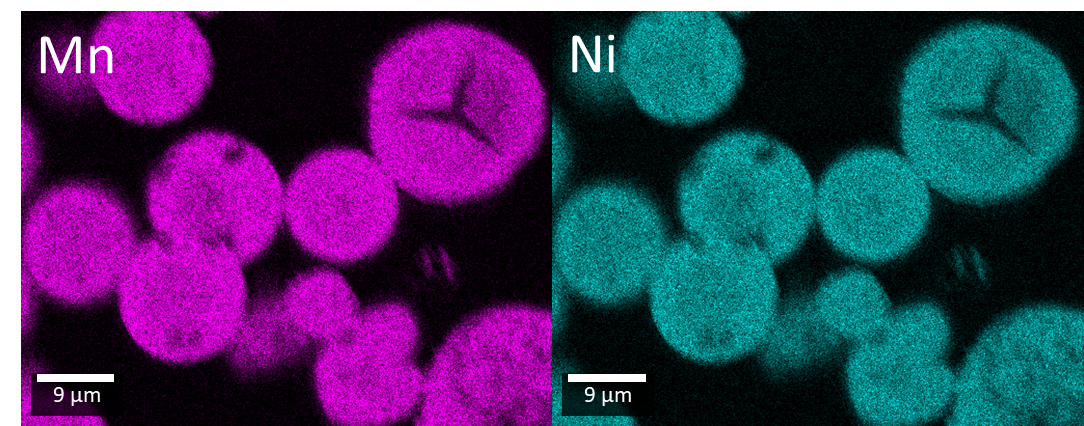
**

Figure S9.1: *Images showing the raw data recorded for the MnK and NiK channels via EDX analysis applied to the d/o-LNMO product. These were used to calculate the images showing the Ni/Mn-ratio in Fig. 6c) by division of the NiK through the MnK channels.*

**S10: Rate capability test plot for the d-, o- and d-o-LNMO modifications**

**
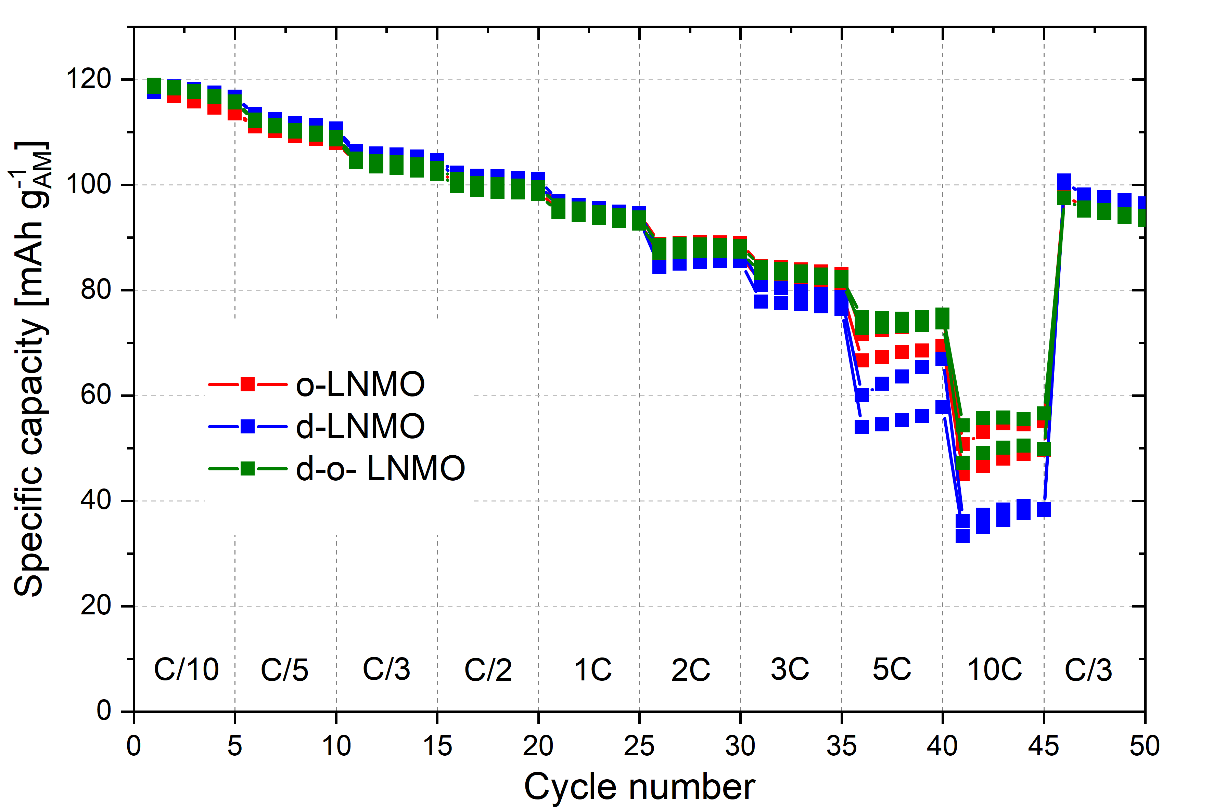
**

Figure S10: *Rate capabilities of the synthesized o-, d- and d-o-LNMO modifications at rates from C/10 to 10C.*

**S11: Cycling performance for the d-, o- and d-o-LNMO**

**
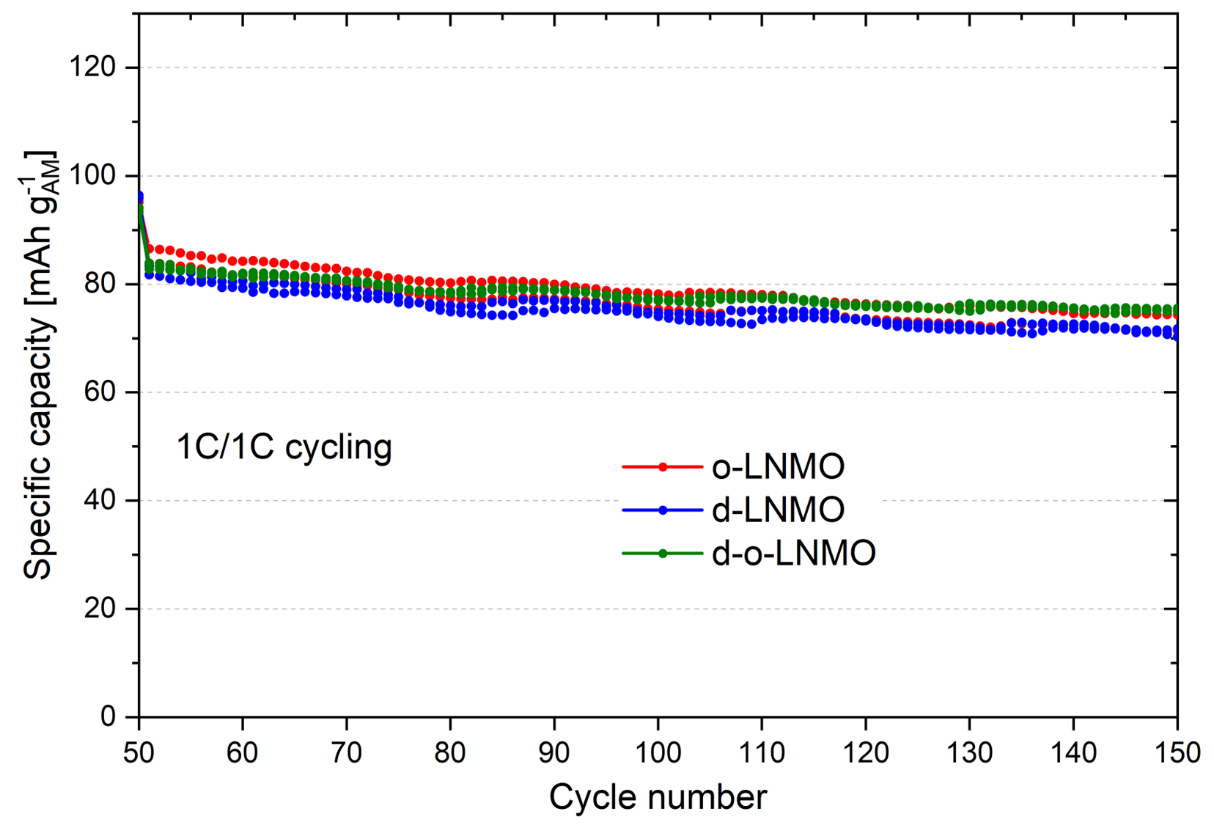
**

Figure S11: *Cycling performance for the d-, o- and d-o-LNMO modifications at a C-rate of 1C (during charge and discharge).*
